# *Brucella*-Induced Downregulation of lncRNA Gm28309 Triggers Macrophages Inflammatory Response Through the miR-3068-5p/NF-κB Pathway

**DOI:** 10.3389/fimmu.2020.581517

**Published:** 2020-12-22

**Authors:** Xingmei Deng, Jia Guo, Zhihua Sun, Laizhen Liu, Tianyi Zhao, Jia Li, Guochao Tang, Hai Zhang, Wenjing Wang, Shuzhu Cao, Dexin Zhu, Tingting Tao, Gang Cao, P. I. Baryshnikov, Chuangfu Chen, Zongsheng Zhao, Lihua Chen, Hui Zhang

**Affiliations:** ^1^ State International Joint Research Center for Animal Health Breeding, College of Animal Science and Technology, Shihezi University, Shihezi, China; ^2^ College of Veterinary Medicine, Xinjiang Agricultural University, Urumqi, China; ^3^ Technology Center, Xinjiang Tianrun Dairy Biological Products Co., Ltd, Urumqi, China; ^4^ Department of Transfusion Medicine, Southern Medical University, Guangzhou, China; ^5^ State Key Laboratory of Agricultural Microbiology, Huazhong Agricultural University, Wuhan, China; ^6^ College of Veterinary, Altai National Agricultural University, Barnaul, Russia; ^7^ College of Chemistry and Molecular Engineering, Qingdao University of Science and Technology, Qingdao, China

**Keywords:** LncRNA Gm28309, *Brucella*, inflammation, miR-3068-5p, NF-κB

## Abstract

**Objectives:**

The underlying mechanism of the inflammatory response against Brucellosis caused by *Brucella* remains poorly understood. This study aimed to determine the role of long non-coding RNAs (lncRNAs) in regulating of inflammatory and anti-*Brucella* responses.

**Materials and methods:**

Microarray analysis was performed to detect differentially expressed lncRNAs in THP-1 cells infected with an S2308 *Brucella* strain. The candidate lncRNAs were screened using bioinformatic analysis and siRNAs; bioinformatic prediction and luciferase reporter assay were also conducted, while inflammatory responses was assessed using RT‐qPCR, western blot, immunofluorescence, ELISA, HE, and immunohistochemistry.

**Results:**

The lncRNA Gm28309 was identified to be involved in regulating inflammation induced by *Brucella.* Gm28309, localized in the cytoplasm, was down-expressed in RAW264.7 cells infected with S2308. Overexpression of Gm28309 or inhibition of miR-3068-5p repressed p65 phosphorylation and reduced NLRP3 inflammasome and IL-1β and IL-18 secretion. Mechanistically, Gm28309 acted as a ceRNA of miR-3068-5p to activate NF-κB pathway by targeting κB-Ras2, an inhibitor of NF-κB signaling. Moreover, the number of intracellular *Brucella* was higher when Gm28309 was overexpressed or when miR-3068-5p or p65 was inhibited. However, these effects were reversed by the miR-3068-5p mimic.

**Conclusions:**

Our study demonstrates, for the first time, that LncRNAs are involved in regulating immune responses during *Brucella* infection, and Gm28309, an lncRNA, plays a crucial role in activating NF-κB/NLRP3 inflammasome signaling pathway.

## Introduction

The Gram-negative facultative intracellular bacteria *Brucella* spp. (*B.* spp.) cause brucellosis, a systemic infectious zoonotic disease. In animals (1), brucellosis leads to abortion, infertility, and lameness, causing serious economic losses. While in humans, (2) *B.* spp. causes symptoms such as undulant fever, endocarditis, arthritis, and osteomyelitis. Brucellosis poses a a serious threat for both livestock and human since *B.* spp. can be transmitted from animals to humansthrough various infection sources, including meat, unpasteurized milk, animal byproducts from infected animals, which lead to economic loss (3) and are public health concerns (4).

It has been reported that the interactions between macrophage cells and *Brucella* alters the macrophage gene expression profile (5). Thus, the investigation of transcriptional and post-transcriptional changes in macrophages may help to further elucidate the anti-*Brucella* immune mechanisms. Although *Brucella* possesses immune subversion mechanisms (6) to evade recognition by toll-like receptors (TLRs) and the immune system activation (7), more than 90% of internalized Brucella are eliminated by the innate immune response (8), indicating that the non-specific inflammatory response against pathogens plays a critical role in controlling *Brucella* infection. Nucleotide-binding oligomerization domain-like receptors (NLRs) are innate cytosolic receptors that can recognize different pathogen-associated molecular patterns (PAMPs) and damage-associated molecular patterns (DAMPs) (9). NLRP3 is the best-characterized inflammasome primarily induced by TLR activation, cytokine stimulation, and other signaling molecules (10). A recent study has demonstrated that *Brucella* ligands activate NLRP3 inflammasomes, leading to infection control (11). The activation of the NLRP3 inflammasome is regulated at the transcriptional level through the NF-κB pathway (12), which induces the synthesis of the pro-inflammatory interleukin pro-IL-1β and increases NLRP3 expression (13).

Non-coding RNAs (ncRNAs), including long non-coding RNAs (lncRNAs) and microRNAs (miRNAs), regulate various innate and adaptive immune processes (14). lncRNAs are defined as transcripts longer than 200 nucleotides lacking an obvious open reading frame (15). Recently, lncRNAs have been shown to regulate gene expression in various physiological and pathological immune processes (16), through a variety of mechanisms, such as behaving as RNA-binding proteins (RBPs), function as decoys, and microRNA sponges. For instance, the lncRNA, Neat1, stabilizes caspase-1 form and increases inflammasomes assembly by binding to pro-caspase-1 (17); NKILA, an NF-κB-interacting lncRNA, inhibits NF-κB activity to regulate the activation-induced cell death of T cell (18). The most well-studied ncRNAs are miRNAs, which during immune response, typically target mRNA transcripts for inflammasome complexes components to regulate inflammation (19). For example, the microRNA miR-223-3p directly targets two components of the NLRP3 inflammasome, namely, caspase-1, and caspase-8 (20), the miR-145a-5p activates the NLRP3 inflammasome by targeting CD137 and NFATc1 (21). Despite these discoveries, a comprehensive view of the role of ncRNAs in the regulation of host–*Brucella* interactions is currently lacking. Furthermore, the lncRNAs-mediated molecular mechanisms underlying the innate immune response against *Brucella* is largely unknown.

## Materials and Methods

### Bacterial Strains and Plasmids

Brucella abortus wild-type strain 2308 (S2308) (The Center of Chinese Disease Prevention and Control, Beijing, China) was cultured in tryptic soy broth (TSB) or tryptic soy ager (TSA) (Difco, MI, USA) at 37°C in 5% CO_2_ incubator, the Brucella strain was manipulated in a biosafety level 3 laboratory. Escherichia coli DH5α (The Center of Chinese Disease Prevention and Control, Beijing, China) was cultured in Luria-Bertani medium, when appropriate, 100 μg/mL of ampicillin or kanamycin was added to the culture medium. pcDNA3.1 (Youbio,Wuhan, China) and pGL3 plasmid (Promega, Beijing, China), and other constructed plasmids were extracted using Endotoxin-free plasmid extraction kit (TianGen, Beijing, China) for cells transfection.

### Cells Culture and Infection

RAW 264.7 and THP-1 cell lines were purchased from Cell Resource Center(Beijing, China) and incubated in the incubator under 37°C and 5% CO_2_ conditions. RAW 264.7 cells were cultured in DMEM (Hyclone) supplemented with 10% fetal bovine serum (Gibco). Prior to infection, RAW 264.7 were seeded to proper culture plates at a density of 10^5^ cells/mL in complete culture medium without penicillin and streptomycin. THP-1 cells were cultured in RPMI-1640 medium (Hyclone) containing 10% fetal bovine serum (Gibco); THP-1 monocytes were differentiated into macrophages with 200 nM PMA (Beyotime, Shanghai, China) for 24 h. All cells were infected with S2308 strain at a MOI of 50. Culture plates were centrifuged at 350 × *g* for 5 min at room temperature and incubated at 37°C for 60 min. After washing twice with PBS, the infected cells were incubated for an additional 45 min in the presence of 50 μg/mL of gentamicin to kill extracellular bacteria. Then, the cultures were placed in fresh DMEM containing 25 μg/mL of gentamicin (defined as time zero) and incubated at 37°C.

### Animal Experiments

The animal experiments were performed according to published methods (22), six-week-old female BALB/c mice (n = 20 per group) were inoculated intraperitoneally either with 200 μL of PBS alone or containing 1×10^6^ CFU of S2308. Every week until 4 weeks post-inoculation, mice (n = 5 per time point per group) were euthanized and their liver and spleen were removed aseptically and used for histopathological observation and immunohistochemistry experiments, the animal experiments were manipulated in a biosafety level 3 laboratory.

### Microarray Analysis of lncRNA and mRNA Expression

Three biological replicates samples of THP-1 cells infected by S2308 at 4 and 24 h were lysed with Trizol reagent (Thermo Fisher Scientific) to extract the total RNA. Microarray hybridization was then conducted according to the standard Arraystar protocols with minor modifications using the Human LncRNA Microarray V3.0 (Arraystar, Rockville, MD, USA). The Agilent Array platform was used for the microarray analysis. Acquired array images were analyzed using the Agilent Feature Extraction software (version 11.0.1.1, Agilent Technologies). Quantile normalization and further data processing were performed using the Agilent GeneSpring GX v11.5 software package (Agilent Technologies). Differentially expressed lncRNAs and mRNAs were identified by performing a Volcano Plot filtering and a Fold Change filtering. Kyoto Encyclopedia of Genes and Genomes (KEGG) and Gene Omnibus (GO) analysis were used to investigate the possible functions of these lncRNAs. The microarray experiment was performed by Capitalbio Technology, Beijing, China.

### Cells Transfection

All plasmids and siRNAs (20 uM/uL) were transfected using the Advanced DNA RNA Transfection Reagent™ (ZETA LIFE, USA) according to the manufacturer’s protocol. Briefly, cells were planted on the cell culture plate one day in advance, the cell confluence degree was up to 60–80% at the time of transfection, plasmid or siRNA was directly mixed with transfection reagent according to 1:1 relationship, then mixed by blowing into a pipette for 10–15 times. Following incubation at room temperature for 10–15 min, the complex was added to the cell culture plates, mix gently, and incubated in the CO_2_ incubator for 24 h. All the siRNAs were designed by Gene Pharma (Shanghai, China), transfection efficiency was measured using qRT-PCR. Details are listed in **Supplementary Table 1**.

### Construction of Vectors

We amplified, using PCR, the full-length sequence of Gm23809 and cloned it into the pcDNA3.1 expression vector to obtain a stable Gm23809 overexpression plasmid [Gm23809 (+)]. The wild-type and mutant fragment of Gm28309 were subcloned into the pGL3 plasmid, and the resulting plasmids were referred to as Gm28309-WT and Gm28309-Mut. Likewise, the 3′ UTR sequence of κB-Ras2 mRNA containing either the predicted wild type miR-3068-5p binding site or its mutated form was cloned into the pGL3 plasmid (κB-Ras2-WT and κB-Ras2-Mut, respectively).

### Immunofluorescence Analysis

The cells were fixed with 4% paraformaldehyde for 30 min at room temperature, following permeabilization with 0.1% Triton X-100 and blocking with 1% BSA in PBS, cells were incubated with Goat polyclonal to NLRP3 (ab4207, 1:100) overnight at 4°C. Cells were then washed three times with PBS, and incubated with dylight488 for fluorescent labeling of Donkey anti-goat IgG-488 (Thermo Fisher Scientific) for an additional hour at 37°C. Finally, images were observed using confocal microscope (Carl Zeiss 510, German) and analyzed using LAS AF Lite (Leica).

### Total, Cytoplasmic, and Nuclear RNA Extraction and Preparation

Cytoplasmic and nuclear RNA fractions were prepared according to published methods (23), with some modifications (24). First, the PBS used in this experiment was supplemented with DEPC, then, cells were collected, washed twice with ice-cold PBS and centrifuged at 1000g for 5 min. The supernatant was removed and the cell pellet was resuspended in 0.3% v/v NP-40-PBS by gently pipetting, and the pellets were placed in ice for 10 min prior to being centrifuged at 1000 × *g* for an additional 5 min. Then, the supernatant was collected separately as the cytoplasmic fraction, and the pellet was washed twice in ice-cold 0.1% NP-40-PBS, centrifuged at 1000 × *g* for 5 min, and kept as the nucleus fraction. For both fractions, RNA was extracted using Trizol reagent (Thermo Fisher Scientifific), following the manufacturer’s protocol. The RNAs were analyzed using qRT-PCR with primer pairs for Gm23809, Actin as cytoplasm control and U1 as nucleus control. Total RNA and miRNA extraction methods are showed in Supplementary materials and methods, all the primer pairs used in study are listed in **Supplementary Table 1**.

### Proteins Extraction and Western Blot

RAW264.7 cells were collected after 24 h of S2308 infection. Cells were lysed in PIPA buffer (Beyotime, Shanghai, China) supplemented with protease and phosphatase inhibitor cocktail (Pierce, Thermo Fisher Scientifific) for 30 min on ice. Lysates were centrifuged at 14,000 × *g* for 30 min and the supernatant was kept as protein extract. The protein content in the extracts was quantified using the BCA protein assay kit (Thermo Fisher Scientific). An equal amount about 15 μg of proteins were separated by 12% SDS-PAGE. The integrated density of protein bands was quantified by Image Lab 3.0 software (Bio-Rad, CA, USA) and normalized against β-actin, used as an internal control. Western blotting was performed using the following primary antibodies (Abcam) and dilutions: rabbit polyclonal anti-TGF-β (ab215715, 1:1,000), rabbit polyclonal anti-Pro-caspase-1 (ab179515, 1:1,000), rabbit polyclonal anti-p65 (ab32536, 1:1,000), and rabbit polyclonal anti-NLRP3 (ab214185, 1:1,000). The primary antibody rabbit polyclonal anti-phospho-p65 (3033T, 1:1,000) was acquired from Cell Signaling Technology, and rabbit polyclonal anti-κB-Ras2 (DF2508, 1:1,000) antibody was obtained from Affinity. The quantitive graph of relative protein expression levels of all proteins were analyzed using image J and showed in **Supplementary Figures**, and β-actin was used as the loading control.

### Bioinformatics Analysis of Gm28309 and Prediction of the Target Gene For miR-3068-5p

Gene information for HOXA10-HOXA9 (Human) and Gm28309 (Mouse) was retrieved from UCSC and Ensembl. The orthologues of human lncRNA HOXA10-HOXA9 in mice were found in a previously published method (25) using the LongMan (http://lncrna.smu.edu.cn) database. The potential connection between Gm28309 and miRNAs were discovered using the LncBase v.2 (http://carolina.imis.athena-innovation.gr/diana_tools/web/index), while the correlation between miR-3068-5p and inflammation genes was screened using the miRDB database (http://www.mirdb.org/index.html).

### Luciferase Reporter Assay

The wild-type and mutant fragment of Gm28309 were subcloned into the pGL3 plasmid, the resulting plasmids were called Gm28309-WT and Gm28309-Mut. Likewise, the 3′ UTR sequence of κB-Ras2 mRNA containing either the predicted wild type miR-3068-5p binding site or its mutated form was cloned into the pGL3 plasmid (κB-Ras2-WT and κB-Ras2-Mut, respectively). miR-3068-5p mimic, inhibitor or negative control were purchased from Gene Pharma (Shanghai, China).

Cells were co-transfected with the appropriate plasmid and oligomers using the Advanced DNA RNA Transfection Reagent™ (ZETA LIFE, USA). Luciferase activity was measured 48 h of transfection using the dual-luciferase reporter assay system (Promega, Madison, WI, USA).

### Interleukins Detection by ELISA

Culture supernatants were harvested from RAW 264.7 infected with S2308 for 24 h, at an MOI of 100:1 in triplicate wells. Moreover, serum samples were obtained from immunized mice 1, 2, 3, and 4 weeks post-immunization as described previously (26). The presence of IL-1β and IL-18 in samples was detected using an ELISA kit (molbio), according to the manufacturer’s instructions.

### Immunohistochemistry (IHC)

Briefly, paraffin-embedded tissues were sliced into 4 μm-thick slices, dewaxed and rehydrated using ethanol at gradual concentrations. Subsequently, antigen retrieval was performed by using the Target Retrieval Solution (Dako, Denmark), according to the manufacturer’s instructions. Sections were treated with 0.3% hydrogen peroxide and then probed with primary antibodies (Abcam) and dilutions overnight:Rabbit polyclonal anti-NLRP3 (ab214185, 1:500), rabbit polyclonal anti-Caspase-1 (ab138483, 1:100), and rabbit monoclonal anti-TGF-β (ab169771, 1:500) and then incubated with secondary antibodies goat anti-rabbit IgG (1:1,000). A 3,39-diaminobenzidine (DAB) substrate kit (Vector Laboratories) was used to detect the proteins.

### Statistical Analysis

Statistical analyses were performed using GraphPad Prism 5 software, one-tailed or t-test was used to determine the differences, the data were expressed as mean values ± standard deviation (SD). *p < 0.05, **p < 0.01, and ***p < 0.001 were considered statistically significant. Each treatment was repeated at least three times.

## Results

### *Brucella* Infection-Induced Inflammatory Response In Vitro and Vivo

**Figure 1:**
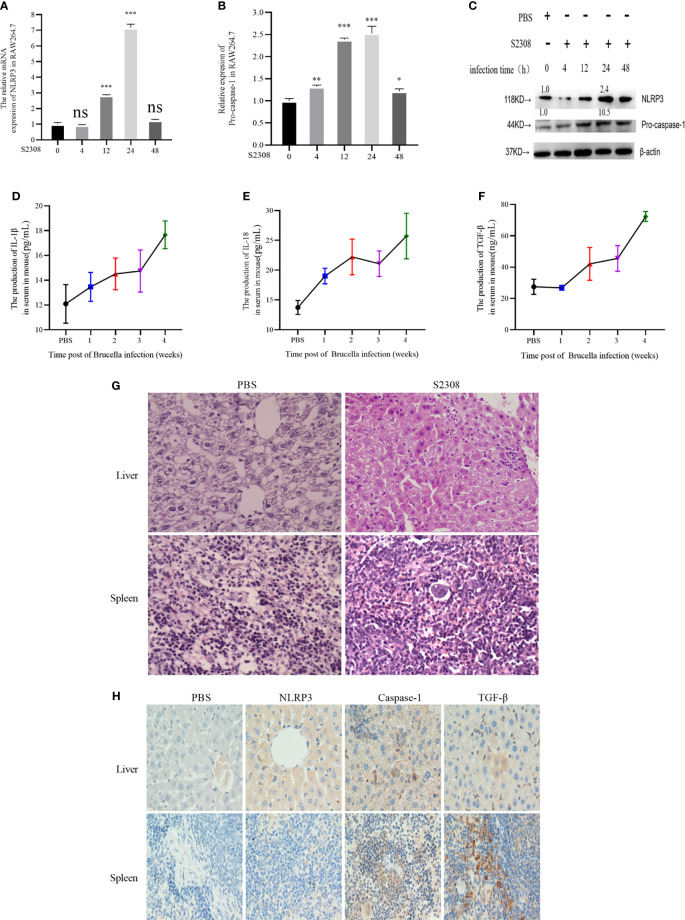
*Brucella* infection-induces an inflammatory response *in vitro* and *vivo*. **(A, B)** The mRNA expression of NLRP3 and Pro-caspase-1 in RAW264.7 cells infected with at 4, 12, 24, and 48 h of S2308 infection using qRT-PCR. **(C)** Protein expression of NLRP3 and Pro-caspase-1 assessed by western blotting. **(D–F)** Expression levels of IL-1β, IL-18 and TGF-β in the blood serum of mice infected by S2308, as detected by ELISA. **(G)** Representative H&E-stained liver and spleen issues of *Brucella*-infected mice. Bar, 80 µm. **(H)** Representative immunohistochemistry of NLRP3 and caspase-1 levels in liver and spleen of *Brucella*-infected mice. Bar 100µm. Data are shown as mean ± SD (n = 3). *p < 0.05, **p < 0.01, ***p < 0.001, one-tailed t-test. ns, not significant.

The innate immune system is essential for detection and elimination of bacterial pathogens, inflammatory response caused by *Brucella* controls infection and be a protective effect in host, inflammasome NLRP3 complex was activated during *Brucella* infection (11). To study inflammatory response of *Brucella* infection *in vitro*, macrophages were infected by S2308 at different time and NLRP3 and Pro-caspase-1 were detected at mRNA (**Figures 1A, B**) and protein levels (**Figure 1C**), our results showed that inflammasome NLRP3 and Pro-caspase-1 were significantly increased, especially at 24 h of infection. Furthermore, in the study *in vivo*, mice were infected with *B. abortus* 2308 strain by intraperitoneal injection (dose 1×10^6^ cells). In parallel, the control group was injected with the same volume of PBS solution. Peripheral blood was collected weekly until 4 weeks following injection. We then measured the levels of the IL-1β, IL-18 inflammatory interleukins, and TGF-β in the serum using ELISA and found a significant increase in all the examined time points compared with the control group (**Figures 1D–F**). Histopathological examination of the liver tissues of the infected mice revealed nuclei dissolution, cell necrosis, and a small number of cells undergoing fatty degeneration. The examination of spleen tissues revealed the presence of endothelial cells in nodules and local tissue congestion (**Figure 1G**). Furthermore, IHC assay showed that NLRP3, Caspase-1, and TGF-β were significantly higher in the infected group (**Figure 1H**). The results demonstrated inflammatory response were activated during Brucella infection in vivo and vitro.

### Differentially Expressed lncRNAs Involved in Inflammatory Responses in THP-1 Cells Infected With *Brucella* S2308

**Figure 2:**
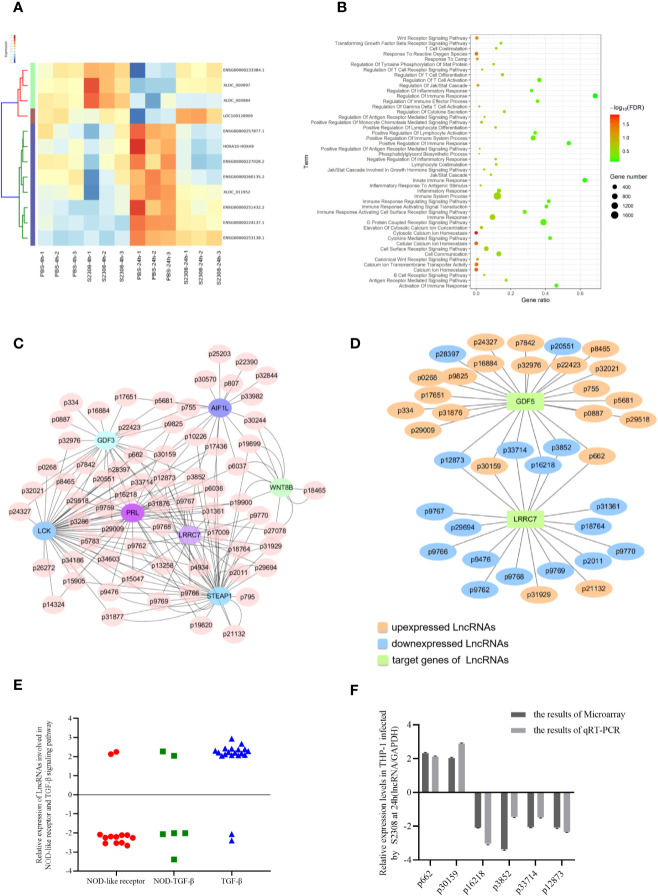
Identification of differentially expressed lncRNAs involved in inflammatory responses in THP-1 cells infected by S2308. **(A)** Partial heatmap showing differentially expressed lncRNAs in THP-1 cells infected by S2308 at 4 and 24 h. **(B)** KEGG enrichment analysis for differentially expressed lncRNAs in THP-1 cells infected by S2308 for 24 h. **(C)** Constructed lncRNA-mRNA correlation network according to the prediction of potential targets using co-expression analysis. **(D)** lncRNAs involved NOD-like receptor pathway and TGF-β signaling pathway. **(E)** Relative expression of the 42 lncRNAs involved in the NOD-like receptor pathway and TGF-beta signaling pathway in THP-1 cells infected by S2308 for 24 h. **(F)** Expression levels of the six lncRNAs involved in inflammation in THP-1 cells infected with S2308 for 24 h, as detected using qRT-PCR and microarray analysis.

To evaluate which lncRNAs are differentially expressed during *Brucella* infection, the total RNA of three biological replicates was extracted from THP-1 cells infected with S2308 at 4 h and 24 h. The lncRNAs were analyzed by microarray, using the Human long non-coding RNA (lncRNA) V 3.0 gene chip. lncRNAs expression change more than two-fold in all the three biological replicates were selected to further study. Compared to the uninfected group (PBS), there were 54 differentially expressed lncRNAs (46 upregulated and 8 downregulated) after 4 h following infection, and 235 differentially expressed lncRNAs (171 upregulated and 64 downregulated) 24 h following infection. The heatmap of partial differentially expressed lncRNAs is shown in **Figure 2A**. We further verify the expressions of partial lncRNAs in THP-1 cells infected by S2308 both 4 and 24 h using qRT-PCR, showing that the same tendency with RNA-seq (**Supplementary Figure 1A**). As the expression of lncRNA after 24 h following infection mimics more closely the physiological changes caused by *Brucella* (27), all the subsequent analyses were performed on the 24 h dataset. The lncRNAs target genes enriched in pathways associated with inflammation and immune response in host were screened out using Gene Omnibus (GO) (**Figure 2B**) and Kyoto Encyclopedia of Genes and Genomes (KEGG) (**supplementary Figure 2B**). To investigate the possible functions of these lncRNAs, we perform a prediction of potential targets using co-expression analysis, a lncRNA-mRNA correlation network was constructed (**Figure 2C**). lncRNAs can act as regulators in innate immunity and antimicrobial defense (28). Therefore, we determined whether the differentially expressed lncRNAs induced by *Brucella* infection could be inflammatory response and antimicrobial defense regulators. To do this, we primarily focused on lncRNAs enriched in the NOD-like receptor signaling pathway and the transforming growth factor β (TGF-β) signaling pathway according to GO and KEGG pathway analysis. The lncRNA-mRNA correlation network for these two pathways is shown in **Figure 2D**. There were 19 differentially expressed lncRNAs associated with the NOD-like receptor signaling pathway and 23 lncRNAs involved in the TGF-β signaling pathway. We then identified 6 lncRNAs, all of which were involved in inflammation (**Figure 2E**): Of the 6 identified lncRNAs, 2 were upregulated, and 4 were downregulated. The expression of these lncRNAs in THP-1 cells was further validated using qRT-PCR (**Figure 2F**).

### Decrease of the lncRNA P33714 Expression Promotes the Secretion of Pro-Inflammatory Cytokines in THP-1 Cells Infected by S2308

**Figure 3:**
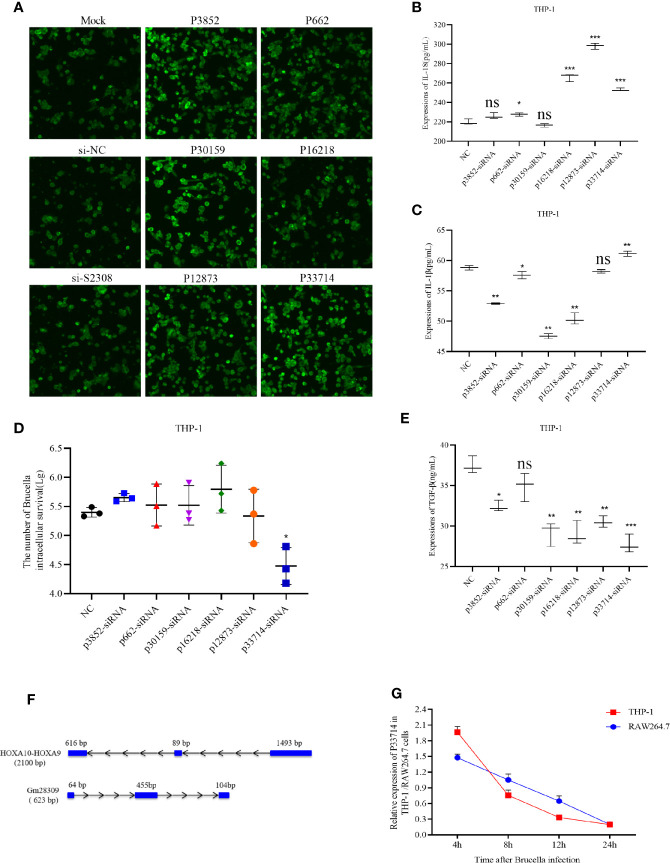
Downregulation of lncRNA P33714 promotes secretion of pro-inflammatory interleukins in THP-1 cells infected by S2308. **(A)** The protein expression of NLRP3 inflammasome by immunofluorescent staining in THP-1 cells transfected with siRNAs of six candidate lncRNAs involved in inflammation. Bar, 450 µm. **(B**, **C**, **E)** Detection, using ELISA of IL-1β, IL-18, and TGF-β secretion in the supernatant of THP-1 cells transfected with the indicated siRNA and infected with S2308 for 24 h. **(D)** The number of *Brucella* intracellular survivors in THP-1 cells transfected with siRNAs of six candidate lncRNAs involved in inflammation. **(F)** Gene structure of the HOXA10-HOXA9 (human) and Gm28309 (mice) transcripts. **(G)** Expression of Gm28309 and p33714 detected using qRT-PCR in THP-1 and RAW264.7 cells infected with S2308 at the indicated time point (4, 8, 12, and 24 h). Data are shown as mean ± SD (n = 3). *p < 0.05, **p < 0.01,***p < 0.001, one-tailed t-test. ns, not significant.

To characterize the role of the six candidate lncRNAs involved in inflammation, we designed three siRNAs for each lncRNA and transfected them into THP-1 cells to detect the expression of the target lncRNA (**Supplementary Table 1**). siRNAs efficiency was screened using qRT-PCR and the most efficient siRNA for each lncRNA (**Supplementary Table 2**) was used for subsequent experiments. The selected siRNAs were transfected into THP-1 cells for 24 h, which were subsequently infected by S2308 for 24 h. Laser confocal results shown that the expression of NLRP3 inflammasome in THP-1 cells was higher in P3852-siRNA, P30159-siRNA, and P33714-siRNA than other groups (**Figure 3A**). Further, the expression of pro-inflammatory interleukin IL-1β decreased significantly upon the knock-down of P3852, P662, P30159, and P16218, but not P33714 (**Figure 3B**). Conversely, IL-18 levels were lower upon knock-down of P662 but higher upon knock-down of P16218, P12873, and P33714 (**Figure 3C**). We found that knock-down of P33714 could inhibit Brucella intracellular survival (**Figure 3D**), and that the expression of TGF-β was lower for all the knock-downs (**Figure 3E**). Collectively when P33714 was knocked-down, IL-1β and IL-18 increased and the number of surviving *Brucella* in THP-1 cells decreased. Based on these results, we focused on the lncRNA P33714 (NR_037940.1), which is a readthrough of the HOXA10-HOXA9 genes. P33714 is located on chromosome 7p15.2 (from nucleotide 27162438 to 27180261). It consists of 2198 nucleotides and 3 exons. A platform created by Jie Lin (25) contains the orthologs of human and mouse lncRNAs annotated in GENCODE, which allows us to search for the orthologous lncRNA of P33714.In this way, we found the P33714 ortholog in mice, called Gm28309 (Ensembl Gene ID: ENSMUSG00000099521.1). Gm28309 is located on chromosome 2 (from nucleotide 74,683,446 to 74,694,194), and is a 623 transcript forward strand with 3 exons (**Figure 3F**). P33714 and Gm28309 expression levels decreased in THP-1 and RAW264.7 cells infected by S2308, respectively. Although they showed a different kinetic expression at 24 h post-infection they showed the same reduced expression levels (**Figure 3G**). Therefore, we suspected that Gm28309 may be involved in the activation of inflammation in host cells in response to *Brucella* infection.

### Gm28309 Overexpression Negatively Regulates the Production of NLRP3 Inflammasome in RAW 264.7 Cells Infected by S2308

**Figure 4:**
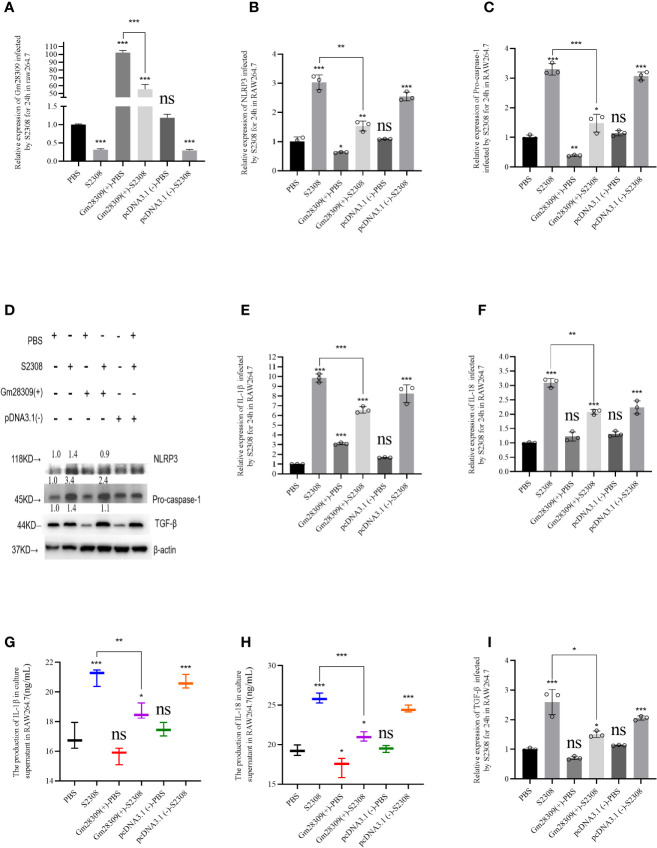
Gm28309 overexpression negatively regulates the production of NLRP3 inflammasome in RAW 264.7 cells infected by S2308. **(A)** Expression of Gm28309 in RAW264.7 cells transfected with the overexpressed plasmid Gm28309-pcDNA3.1, as detected using qRT-PCR. **(B**, **C**, **E**, **F**, **I)**. The relative expression of NLRP3, Pro-caspase-1, IL-1β, IL-18, and TGF-β when overexpressed Gm28309 in RAW264.7 cells, as detected using qRT-PCR. **(D)** expression of NLRP3, Pro-caspase-1, and TGF-β as detected using western blotting when overexpressed Gm28309 in RAW264.7 cells. **(G**, **H)** IL-1β and IL-18 levels in the supernatants in RAW264.7 cells transfected with the overexpressed plasmid Gm28309-pcDNA3.1 as detected usjing ELISA. Data shown are mean ± SD (n = 3). *p < 0.05, **p < 0.01,***p < 0.001, one-tailed t-test. ns, not significant.

Among all the NLR inflammasome complexes, the NLRP3 inflammasome is the best-characterized and is a crucial signaling node controlling the maturation of two proinflammatory interleukins belonging to the (IL)-1 family: IL-1β and IL-18 (9). To further characterize the role of P33714 in inflammation, we overexpressed Gm28309 in RAW264.7 cells and detected, using qRT-PCR, the expression levels of NLRP3 inflammasome components, such as NLRP3 and Caspase-1 in RAW264.7 cells overexpressing Gm28309. Results showed that the expression plasmid for Gm28309 (Gm28309(+) significantly increased Gm28309 expression levels when transfected into RAW264.7 cells, compared with mock-transfected cells and cells transfected with an empty pCDNA3.1 (**Figure 4A**). Overexpression of Gm28309 reduced the formation of the NLRP3 inflammasome, decreasing both the mRNA (**Figures 4B, C**) and protein (**Figure 4D**) levels of NLRP3 and pro-caspase-1. Furthermore, mRNA levels of factors activated by the NLRP3 inflammasome (IL-1β and IL-18) were significantly decreased in Gm28309 overexpressed cells, but higher in S2308 infection group (**Figures 4E, F**), as well as protein secretion levels detected using ELISA (**Figures 4G, H**). In addition, we observed an increase in TGF-β expression upon S2308 infection, which was attenuated in cells overexpressing Gm28309 (**Figures 4D, I**). Collectively, our results indicate that Gm28309 expression was decreased in RAW264.7 infected by S2308 whereas overexpression of Gm28309 reduces the production of the NLRP3 inflammasome.

### Gm28309 Sequesters miR-3068-5p to Activate the NF-κB Signaling Pathway

**Figure 5:**
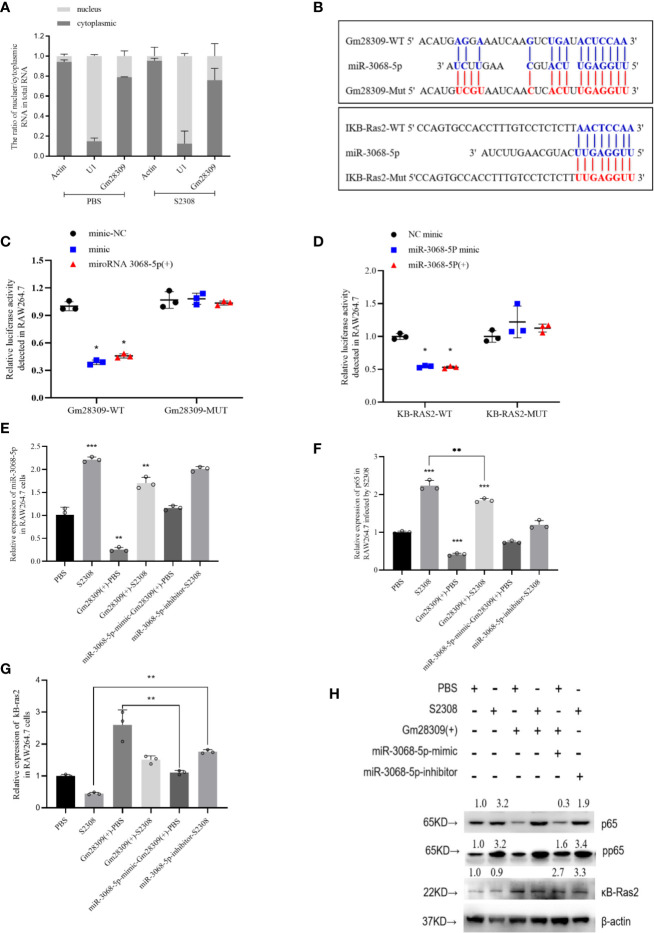
Gm28309 sponges miR-3068-5p to repress the NF-κB signaling pathway. **(A)** Subcellular localization of Gm28309 as determined using qRT-PCR on subcellular fractions. **(B)** Predicted Gm28309 binding and mutant sites on miR-3068-5p and κB-Ras2. **(C**, **D)** Luciferase reporter assays of Gm28309 and κB-Ras2 in response to miR-3068-p5 or its mimic. **(E**–**G)** Expression of miR-3068-5p,p65 and κB-Ras2 in RAW264.7 cells treated with miR-3068-p5 mimic, inhibitor or overexpressed Gm28309 as detected using qRT-PCR. **(H)** Expression of κB-Ras2, p65 and its phosphorylation form in RAW264.7 cells treated with in RAW264.7 cells treated with miR-3068-p5 mimic, inhibitor or overexpressed Gm28309, as determined western blotting on the NF-κB signaling pathway. Data are shown as mean ± SD (n = 3). *p < 0.05, **p < 0.01,***p < 0.001, one-tailed t-test (ordinary one way ANOVA).

lncRNAs can perform various functions depending on their subcellular location (29). If in nucleus, they are primarily involved in transcription and chromatin remodeling, while in the cytoplasm, they participate in gene regulation by forming complexes with specific proteins (30). We analyzed the cellular localization of Gm28309 by performing qRT-PCR of RAW264.7 subcellular fractions. We found that Gm28309 is preferentially localized in the cytoplasm rather than in the nucleus (**Figure 5A**), suggesting a potential role for Gm28309 as a competing endogenous RNA (ceRNA). We speculated that Gm28309 might target factors involved in the NF-κB pathway, a well‐known inflammation-associated signaling pathway (12). Hence, we performed bioinformatics analysis and we detected a potential targets: miR-3068-5p whose target is κB-Ras2 (**Figure 5B**). κB-Ras2 (*NKIRAS2*) is an IκBβ-interacting protein that could inhibit NF-κB activation (31), Moreover, κB-Ras2 knock-out mice show an enhanced IκBβ-dependent inflammatory response (32). Site-directed mutation at site was used to construct the mutant forms of Gm28309 and κB-Ras2 (**Figure 5B**), and the wild-type or mutant forms of Gm28309 and κB-Ras2 were cloned to pGL3 plasmid respectively. The result of luciferase assays showed a decrease of luciferase activity in the presence of miR-3068-5p overexpression, while their mutant forms were not affected (**Figures 5C, D**). Moreover, qRT-PCR results showed that, in RAW264.7 cells infected with S2308, the decreased expression of Gm28309 is accompanied by an increased expression of miR-3068-5p and the NF-κB p65 subunit; in contrast, Gm28309 overexpression decreased levels of miR-3068-5p and p65 levels (**Figures 5E, F**), but increased expression of κB-Ras2 (**Figures 5G, H**). Furthermore, when we treated RAW264.7 cells with an miR-3068-5p inhibitor, we observed a decrease in p65 phosphorylation levels (**Figures 4F, H**) but a significant increase in κB-Ras2 (**Figures 5G, H**), compared with that in cells infected by S2308. These results indicated that, in normal conditions, Gm28309 act as a ceRNA by binding miR-3068-p5 molecules, preventing κB-Ras2 mRNA degradation and ultimately the suppression of the NF-κB pathway. Upon *Brucella* infection, the decreased levels of Gm28309 enable the degradation of κB-Ras2 mRNA by releasing miR-3068-p5 and the activation of the NF-κB pathway.

### *Brucella* Activates the NLRP3 Inflammasome Through the Gm28309–miR3068-5p–NF-κB Regulatory Axis *In Vitro*

**Figure 6:**
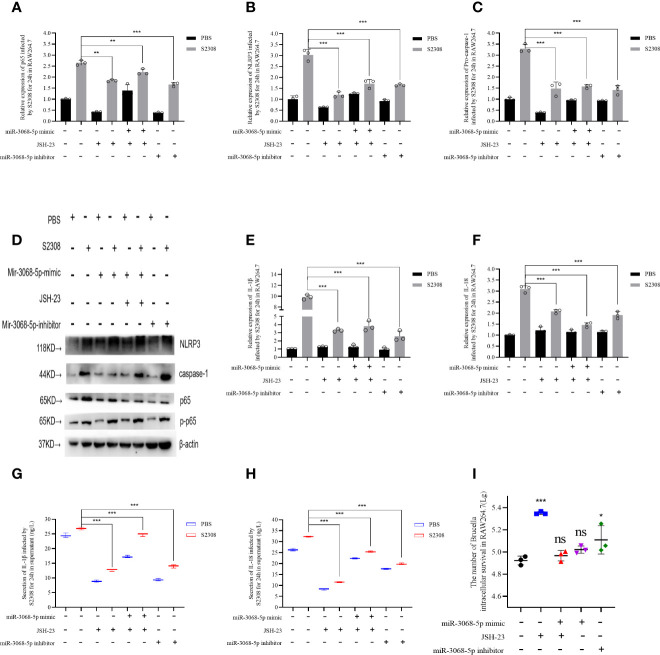
*Brucella* activated NLRP3 inflammasome by Gm28309–miR3068-5p –NF-κB regulatory axis *in vitro*. **(A**–**E)** Expression levels of p65, NLRP3 inflammasome, IL-1β, and IL-18 in RAW264.7 cells transfected with either a miR-3068 inhibitor or its mimic, with or without the specific p65 inhibitor JSH-23, as determined using qRT-PCR. **(F**, **G)** detection of IL-1β and IL-18 from supernatants of the cell treated as in **(A–E)**, as detected using ELISA. **(H)** NLRP3 inflammasome and p65 protein levels, as detected using western blot. **(I)** The number of *Brucella* intracellular survivors. Data are shown as mean ± SD (n = 3). *p < 0.05, **p < 0.01,***p < 0.001, one-tailed t-test. ns, not significant.

We further explored the relationship between miR-3068-5p and NF-κB/NLRP3 inflammasome signaling pathway using a specific small-molecule p65 inhibitor, JSH-23. Data showed that the overexpression of miR-3068-5p could significantly increase the expression of p65 and components of the NLRP3 inflammasome, as determined using qRT-PCR (**Figures 6 A–C**) and western blot (**Figure 6D**). However, cells treated with 300 μM-specific p65 inhibitor JSH-23 for 24 h (33) was observed a decrease in the expression of p65 and components of the NLRP3 inflammasome. Moreover, we found that the expression of the NLRP3 inflammasome was higher when miR-3068-5p was overexpressed in the presence of the p65 inhibitor both at the mRNA level (**Figures 6B, C**) and the protein levels (**Figure 6D**), as well as the secretion of IL-1β and IL-18 (**Figures 6E–H**). Furthermore, the number of intracellular *Brucella* was higher when p65 was inhibited (**Figure 6I**). Therefore, our results demonstrated that Gm28309 has an important role in the NF-κB/NLRP3 inflammasome pathway through its binding to miR-3068-5p.

## Discussion

In this study, we found that the expression profiles of lncRNA and mRNA in macrophages infected with *B. abortus* for 4 and 24 h were significantly altered compared to normal cells. This observation was consistent with what has been previously in human macrophages infected by *Mycobacterium tuberculosis* (34) and *Listeria monocytogenes* (35). Moreover, it is worth noting that more lncRNAs were differentially expressed after 24 h of infection compared to 4 h of infection (235 vs. 54, respectively). These results are consistent with the idea that *Brucella*, which is an intracellular pathogen, use the IV secretion system (T4SS) to deliver bacterial effectors that modulate of the expression of lncRNAs and mRNAs, to alter host functions (36), thereby escaping neutralization and replicate in the Brucella containing vacuole (BCV) (37).

Recognition of *Brucella* PAMPs by different TLRs (38) triggers a signaling cascade to activate NF-κB factors (39), as well as the NLRP3 inflammasome, to control the initial response against *Brucella* (40). The activation of NF-κB is has previously been reported to be correlated with an effective inflammatory response to *B. abortus* infections (41). The role of lncRNAs and miRNAs during the pathogenesis of *Brucella* infection remains poorly understood. In this study, we identified a lncRNA, P33714, as a regulator of the inflammatory response. The expression of P33714 and its murine ortholog Gm28309 expression is decreased in THP-1 and RAW264.7 cells infected by S2308. Moreover, we discovered that Gm28309 negatively regulates the production of NLRP3 inflammasome in RAW 264.7 cells. Collectively, our results suggest a key role for lncRNA Gm28309 in the control of the host response against *Brucella* infection.

**Figure 7:**
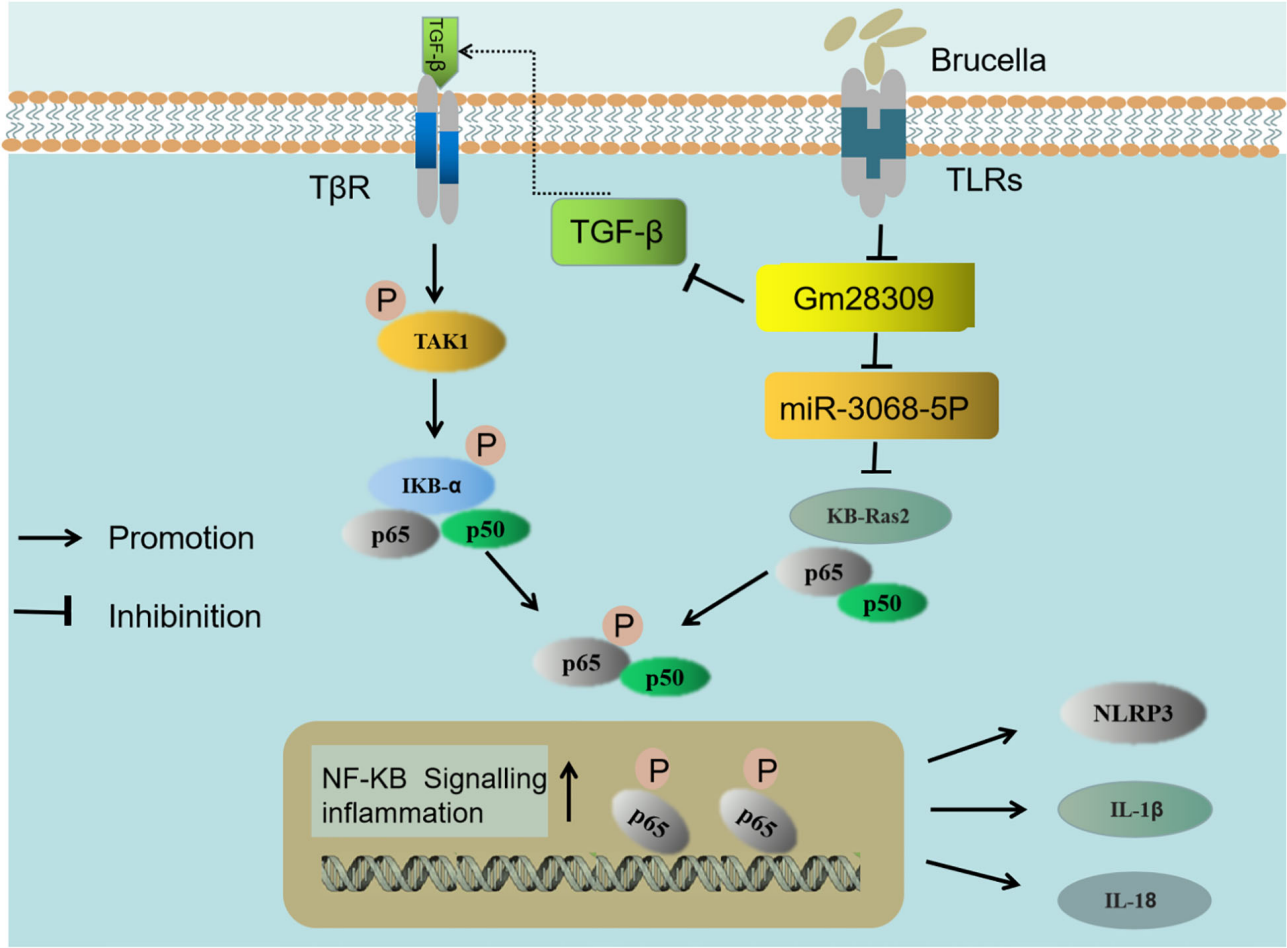
Schematic representation of the proposed model of Gm28309 role in inflammation response. The recognition of *Brucella* by TLRs located on the host cell membrane repressed the expression of lncRNA Gm28309. The decrease of Gm28309 has two effects, both resulting in the activation of the NF-κB pathway: On one side, expression of TGF-β is promoted, leading to a cascade of TAK1 and IKK kinases promoting p65 phosphorylation. On the other side, miR-3068-5p, which is normally sponged by Gm28309, is released in the cytoplasm, where it targets κB-Ras2 for degradation, thereby inducing p65 phosphorylation. The activation of the NF-κB pathway promotes the assembly of the NLRP3 inflammasome and the secretion of IL-1β and IL-18 to intracellular bacterial infection.

It has previously been reported that the expression of miRNAs is significantly altered in RAW264.7 macrophage cells (42), CD4+ T cells (43), and CD8+ T cells (44) upon *Brucella* infection. We found that Gm28309 was primarily localized in the cytoplasm, suggesting a role as a miRNA sponge. Bioinformatics analysis revealed that Gm28309 has predicted binding sites for miR-3068-5p and κB-Ras2. κB-Ras2 is an indirect inhibitor of the NF-κB transcription factor through its binding to IκB proteins. The binding between κB-Ras2 and IκB decrease IκB rate of degradation, blocking NF-κB activation (32). Mechanically, our results suggest a model in which Gm28309 acts as a ceRNA to sequester miR-3068-5p, blocking the degradation of κB-Ras2, which in turn inhibits the NF-κB pathway (**Figure 7**).

TGF-β is a pleiotropic cytokine that is involved in osteoarthritis (45). Inhibition of TGF-β signaling protects adult knee joints in mice against the development of osteoarthritis (46). Interestingly, arthritis is the classical brucellosis symptom, both in humans and animals (47). Consistent with this, our data showed that the expression of TGF-β was higher following *Brucella* infection, both *in vivo* and *in vitro.* We found that TGF-β is decreased while Gm28309 was overexpressed during *Brucella* infection, therefore, the downregulation of Gm28309 upon *Brucella* recognition by TLRs promotes the production of TGF-β to activate the NF-κB signaling and trigger the inflammatory response (**Figure 7**). Further investigations are warranted to elucidate the molecular mechanism underlying Gm28309 negative regulation of TGF-β expression.

Collectively, our results suggest a model in which the recognition of *Brucella* by TLRs located on the host cell membrane represses the expression of lncRNA Gm28309. The decrease of Gm28309 has two effects, both resulting in the activation of the NF-κB pathway: On one side, expression of TGF-β is promoted, leading to a cascade of TAK1 and IKK kinases promoting p65 phosphorylation; while on the other, miR-3068-5p, which is normally sponged by Gm28309, is released into the cytoplasm, where it targets κB-Ras2 for degradation, thereby inducing p65 phosphorylation. The activation of the NF-κB pathway promotes the assembly of the NLRP3 inflammasome and the secretion of IL-1β and IL-18 to intracellular bacterial infection (**Figure 7**) (48). Future studies need to investigate in the future the role of lncRNA-mediated regulation of transcription factors, RNA-RNA interactions, and post-transcriptional regulatory networks on the modulation of the host defense against pathogens immune subversion mechanisms.

## Data Availability Statement

The raw data supporting the conclusions of this article will be made available by the authors, without undue reservation.

## Ethics Statement

The animal study was reviewed and approved by the Animal Health Committee of Shihezi University.

## Author Contributions

XD, HuZ, and LC performed study design. XD, JG, and ZS performed acquisition and collection of data. LL, TZ, and GT involved in contribution of analysis and interpretation of data. RL, SZ, and PB performed bioinformatic analysis. HaZ, WW, and GC provided guidance for analytical tools and performed bioinformatic analysis. DZ and TT prepared figures and reagents. CC and ZZ gave the approval of the final version to be published. HuZ and XD were involved in manuscript preparation. All authors contributed to the article and approved the submitted version.

## Funding

This work was supported by grants from the National Key Research and Development Program of China (grant no. 2017YFD0500304), the National Natural Science Foundation of China (grant nos. 3207190726, 31860691, and 31602080), the International Science and Technology Cooperation Promotion Plan (grant nos. 2015DFR31110 and GJHZ201709), the Training Program for Excellent Young Teachers Colleges and Universities of Corps (grant no. CZ027202), and the Youth Science and Technology Innovation Leading Talent Program of Corps (grant no. 2017CB002).

## Conflict of Interest

GT was employed by Dairy Biological Products Co., Ltd.

The remaining authors declare that the research was conducted in the absence of any commercial or financial relationships that could be construed as a potential conflict of interest.
