# Corrigendum: *Brucella*-Induced Downregulation of lncRNA Gm28309 Triggers Macrophages Inflammatory Response Through the miR-3068-5p/NF-κB Pathway

**DOI:** 10.3389/fimmu.2021.805275

**Published:** 2021-12-13

**Authors:** Xingmei Deng, Jia Guo, Zhihua Sun, Laizhen Liu, Tianyi Zhao, Jia Li, Guochao Tang, Hai Zhang, Wenjing Wang, Shuzhu Cao, Dexin Zhu, Tingting Tao, Gang Cao, P. I. Baryshnikov, Chuangfu Chen, Zongsheng Zhao, Lihua Chen, Hui Zhang

**Affiliations:** ^1^State International Joint Research Center for Animal Health Breeding, College of Animal Science and Technology, Shihezi University, Shihezi, China; ^2^College of Veterinary Medicine, Xinjiang Agricultural University, Urumqi, China; ^3^Technology Center, Xinjiang Tianrun Dairy Biological Products Co., Ltd, Urumqi, China; ^4^Department of Transfusion Medicine, Southern Medical University, Guangzhou, China; ^5^State Key Laboratory of Agricultural Microbiology, Huazhong Agricultural University, Wuhan, China; ^6^College of Veterinary, Altai National Agricultural University, Barnaul, Russia; ^7^College of Chemistry and Molecular Engineering, Qingdao University of Science and Technology, Qingdao, China

**Keywords:** LncRNA Gm28309, *Brucella*, inflammation, miR-3068-5p, NF-κB

In the original article, there was an error in **Figure 1** as published. During the preparation of **Figure 1D** and **Figure 1F** after reviewing, we inadvertently duplicated **Figure 1D** and used it as **Figure 1F** in **Figure 1**. The corrected **Figure 1** appears below.

**Figure 1:**
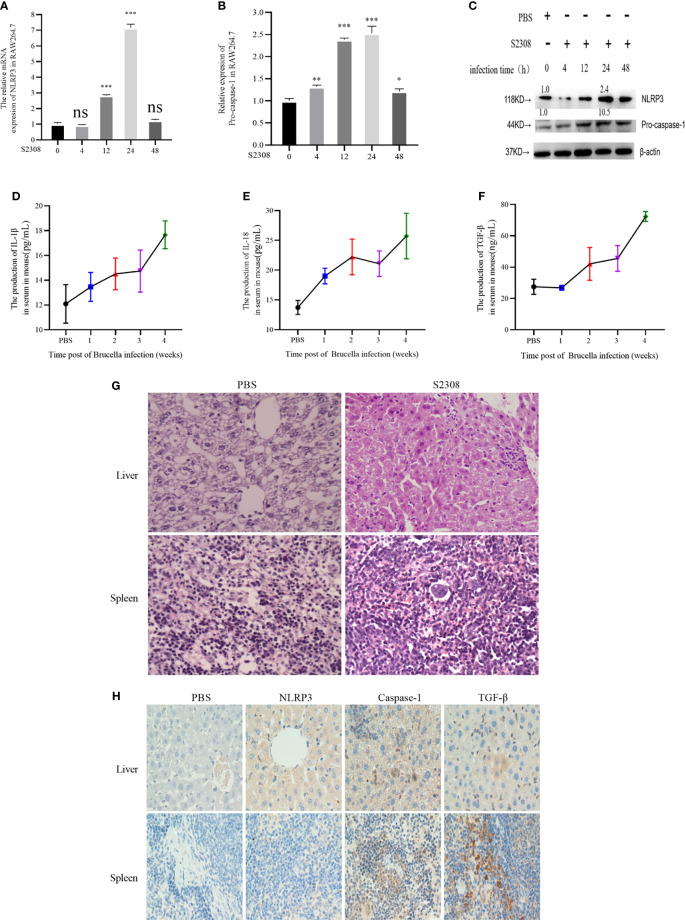
Brucella infection-induces an inflammatory response in vitro and vivo. **(A, B)** The mRNA expression of NLRP3 and Pro-caspase-1 in RAW264.7 cells infected with at 4, 12, 24, and 48 h of S2308 infection using qRT-PCR. **(C)** Protein expression of NLRP3 and Pro-caspase-1 assessed by western blotting. **(D–F)** Expression levels of IL-1β, IL-18 and TGF-β in the blood serum of mice infected by S2308, as detected by ELISA. **(G)** Representative H&E-stained liver and spleen issues of Brucella-infected mice. Bar, 80 μm. **(H)** Representative immunohistochemistry of NLRP3 and caspase-1 levels in liver and spleen of Brucella-infected mice. Bar 100μm. Data are shown as mean ± SD (n = 3). *p < 0.05, **p < 0.01, ***p < 0.001, one-tailed t-test. ns, not significant.

The authors apologize for this error and state that this does not change the scientific conclusions of the article in any way. The original article has been updated.

## Publisher’s Note

All claims expressed in this article are solely those of the authors and do not necessarily represent those of their affiliated organizations, or those of the publisher, the editors and the reviewers. Any product that may be evaluated in this article, or claim that may be made by its manufacturer, is not guaranteed or endorsed by the publisher.

