# Supplementary figures and images for: Brucella-Induced Downregulation of lncRNA Gm28309 Triggers Macrophages Inflammatory Response Through the miR-3068-5p/NF-κB Pathway
Source: Front Immunol. 2020 Dec 22;11:581517. doi: 10.3389/fimmu.2020.581517 (PMC7784117; doi:10.3389/fimmu.2020.581517)

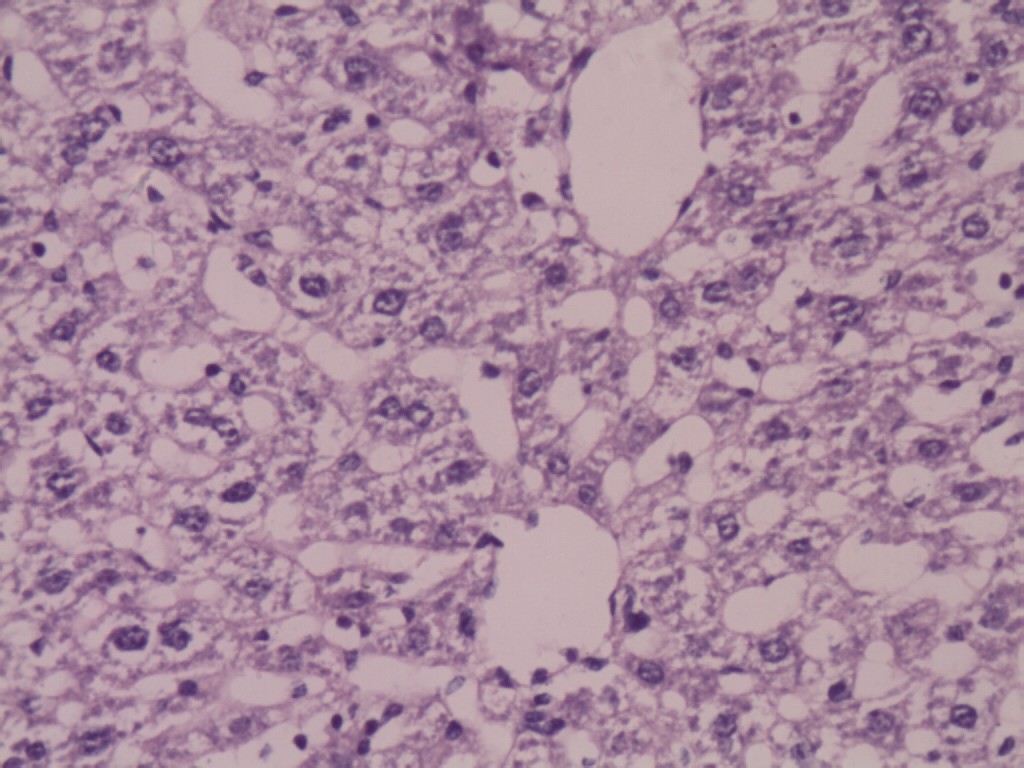

Supplement: Supplementary file 1 [file DataSheet_1.zip › The original images---manuscript 581517/Histopathological original images/Liver-HE 40x in mouse in PBS Group.JPG]

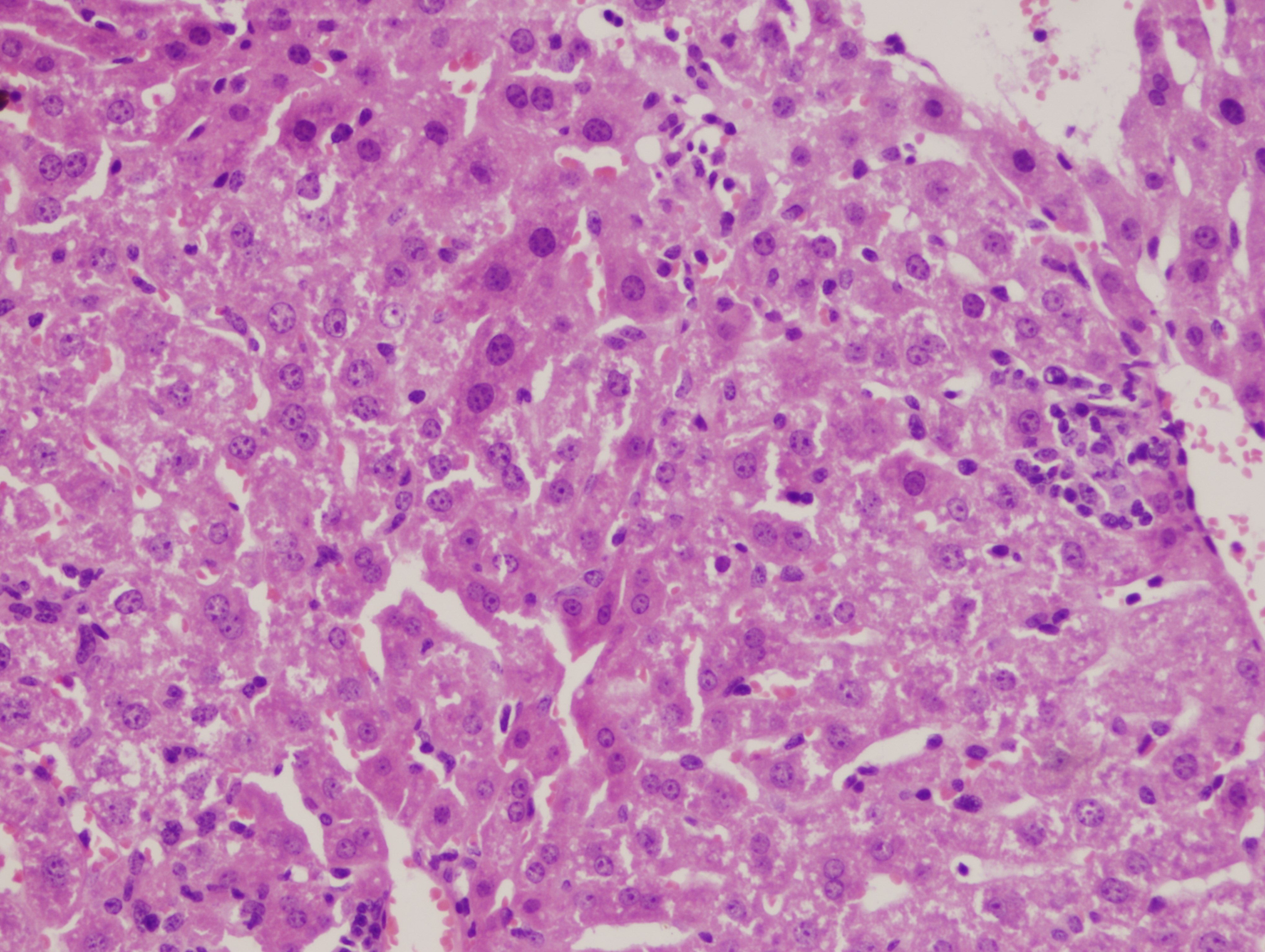

Supplement: Supplementary file 1 [file DataSheet_1.zip › The original images---manuscript 581517/Histopathological original images/Liver-HE 40x in mouse infected by S2308.jpg]

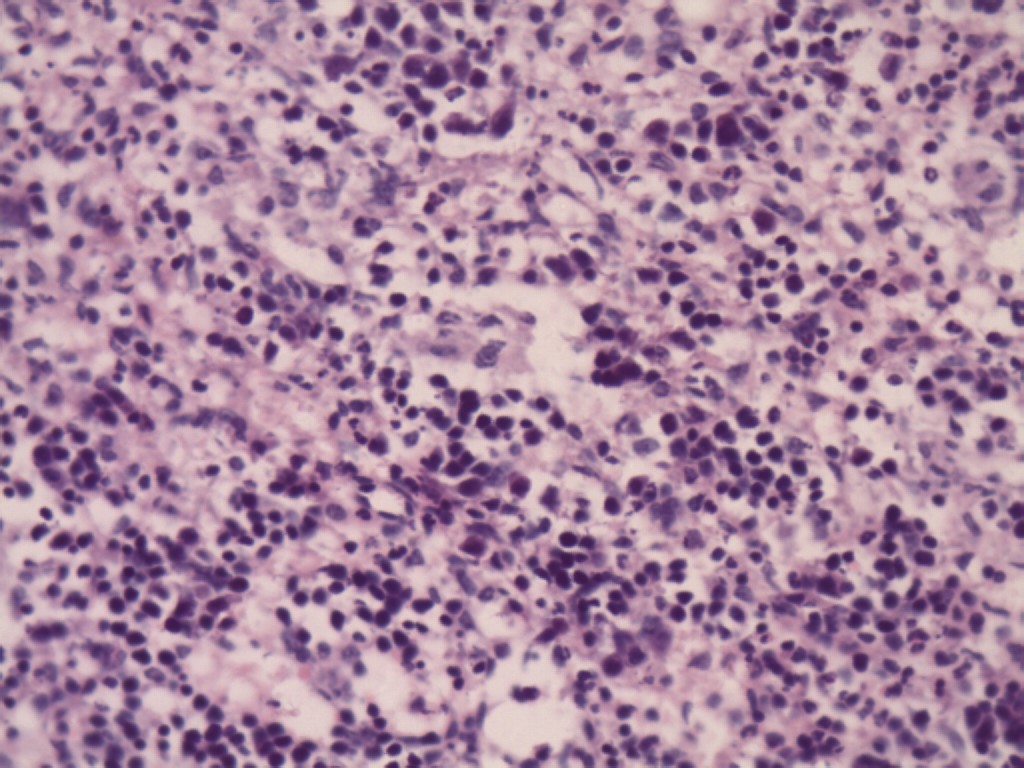

Supplement: Supplementary file 1 [file DataSheet_1.zip › The original images---manuscript 581517/Histopathological original images/Spleen-HE 40x in mouse in PBS Group.JPG]

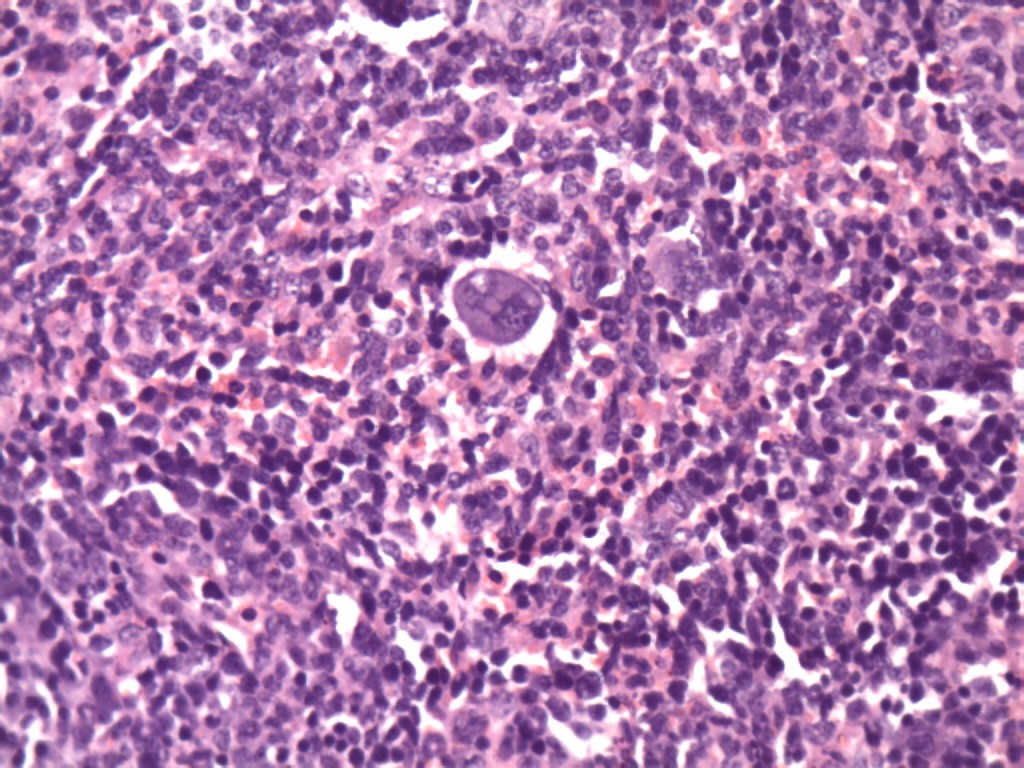

Supplement: Supplementary file 1 [file DataSheet_1.zip › The original images---manuscript 581517/Histopathological original images/Spleen-HE 40x in mouse infected by S2308.JPG]

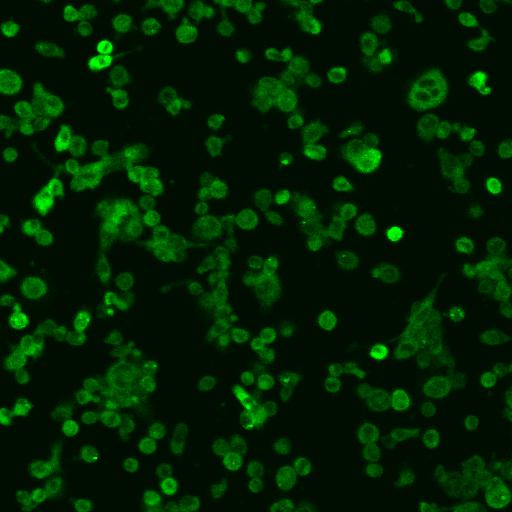

Supplement: Supplementary file 1 [file DataSheet_1.zip › The original images---manuscript 581517/Immunofluorescence original images/P12873-siRNA in THP-1 cell.jpg]

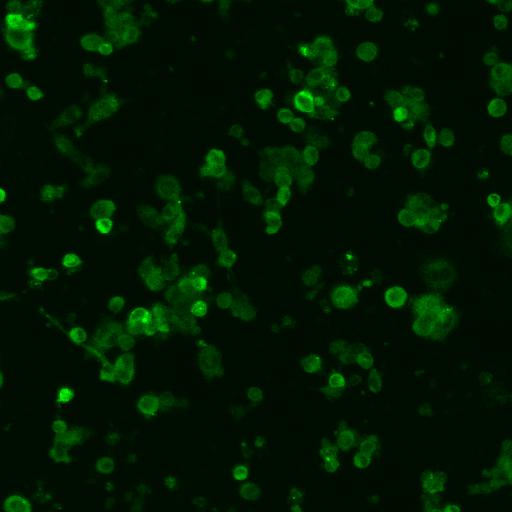

Supplement: Supplementary file 1 [file DataSheet_1.zip › The original images---manuscript 581517/Immunofluorescence original images/P16218-siRNA in THP-1 cell.jpg]

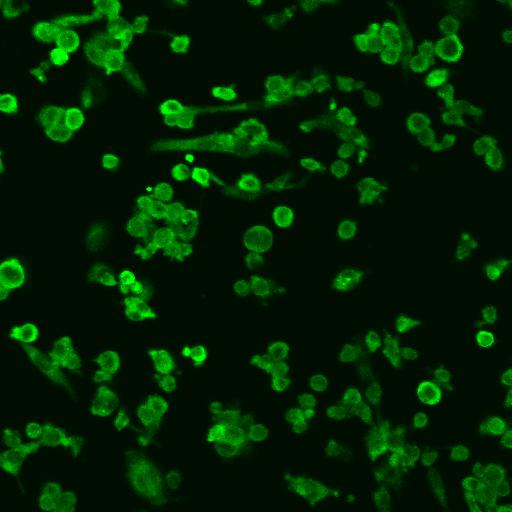

Supplement: Supplementary file 1 [file DataSheet_1.zip › The original images---manuscript 581517/Immunofluorescence original images/P30159-siRNA in THP-1 cell.jpg]

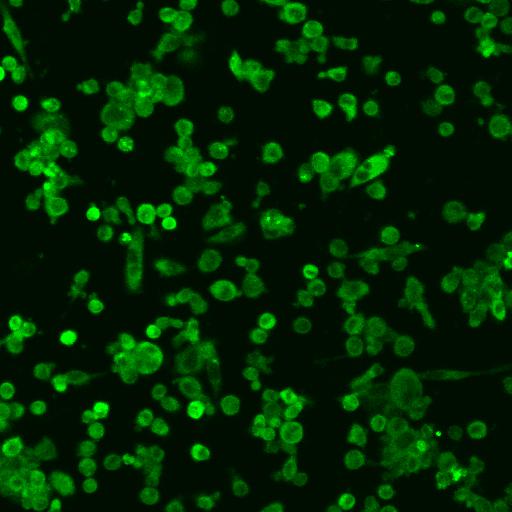

Supplement: Supplementary file 1 [file DataSheet_1.zip › The original images---manuscript 581517/Immunofluorescence original images/P33714-siRNA in THP-1 cell.jpg]

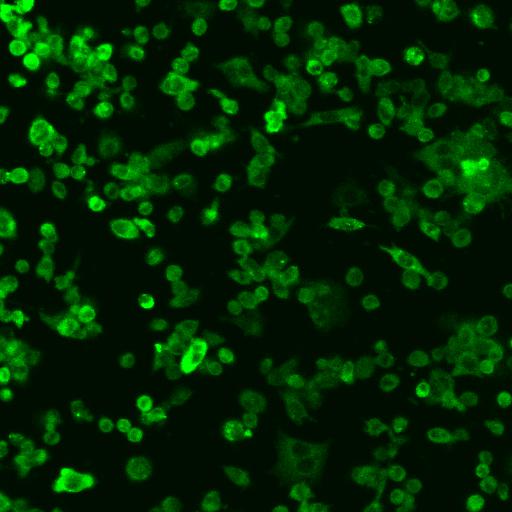

Supplement: Supplementary file 1 [file DataSheet_1.zip › The original images---manuscript 581517/Immunofluorescence original images/P3852-siRNA in THP-1 cell.jpg]

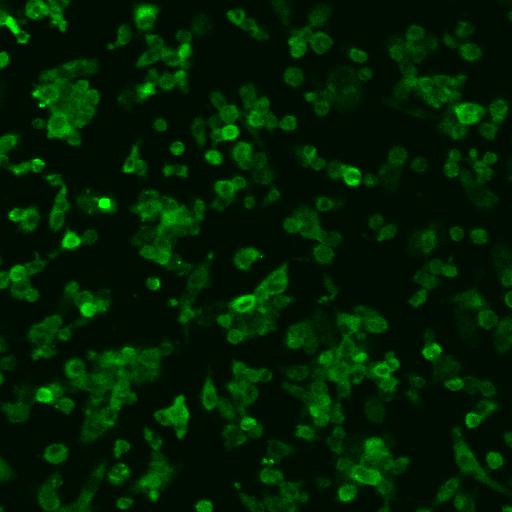

Supplement: Supplementary file 1 [file DataSheet_1.zip › The original images---manuscript 581517/Immunofluorescence original images/P662-siRNA in THP-1 cell.jpg]

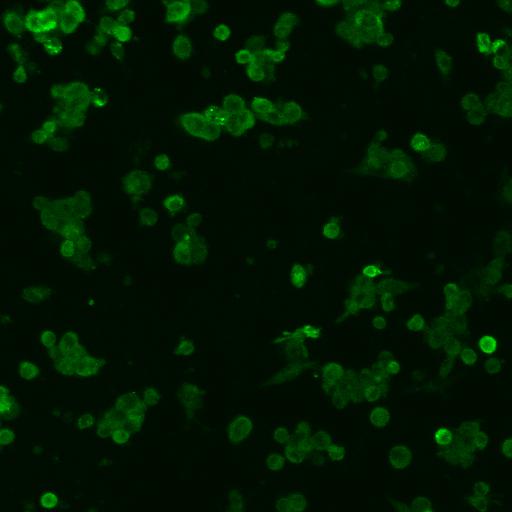

Supplement: Supplementary file 1 [file DataSheet_1.zip › The original images---manuscript 581517/Immunofluorescence original images/mock THP-1 cell.jpg]

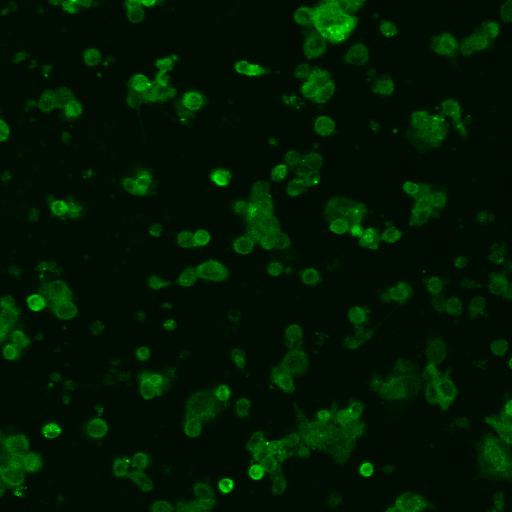

Supplement: Supplementary file 1 [file DataSheet_1.zip › The original images---manuscript 581517/Immunofluorescence original images/siNC-2308.jpg]

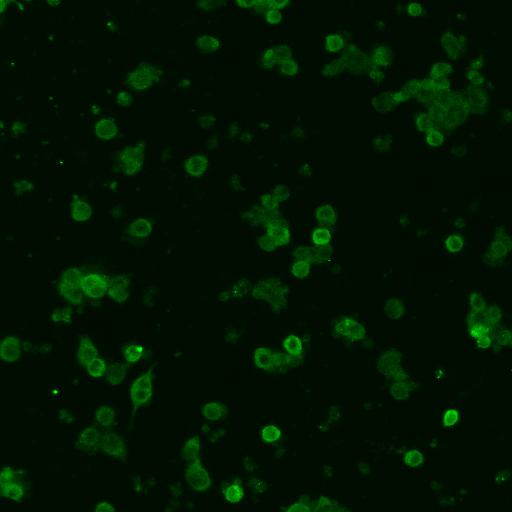

Supplement: Supplementary file 1 [file DataSheet_1.zip › The original images---manuscript 581517/Immunofluorescence original images/siNC-PBS.jpg]

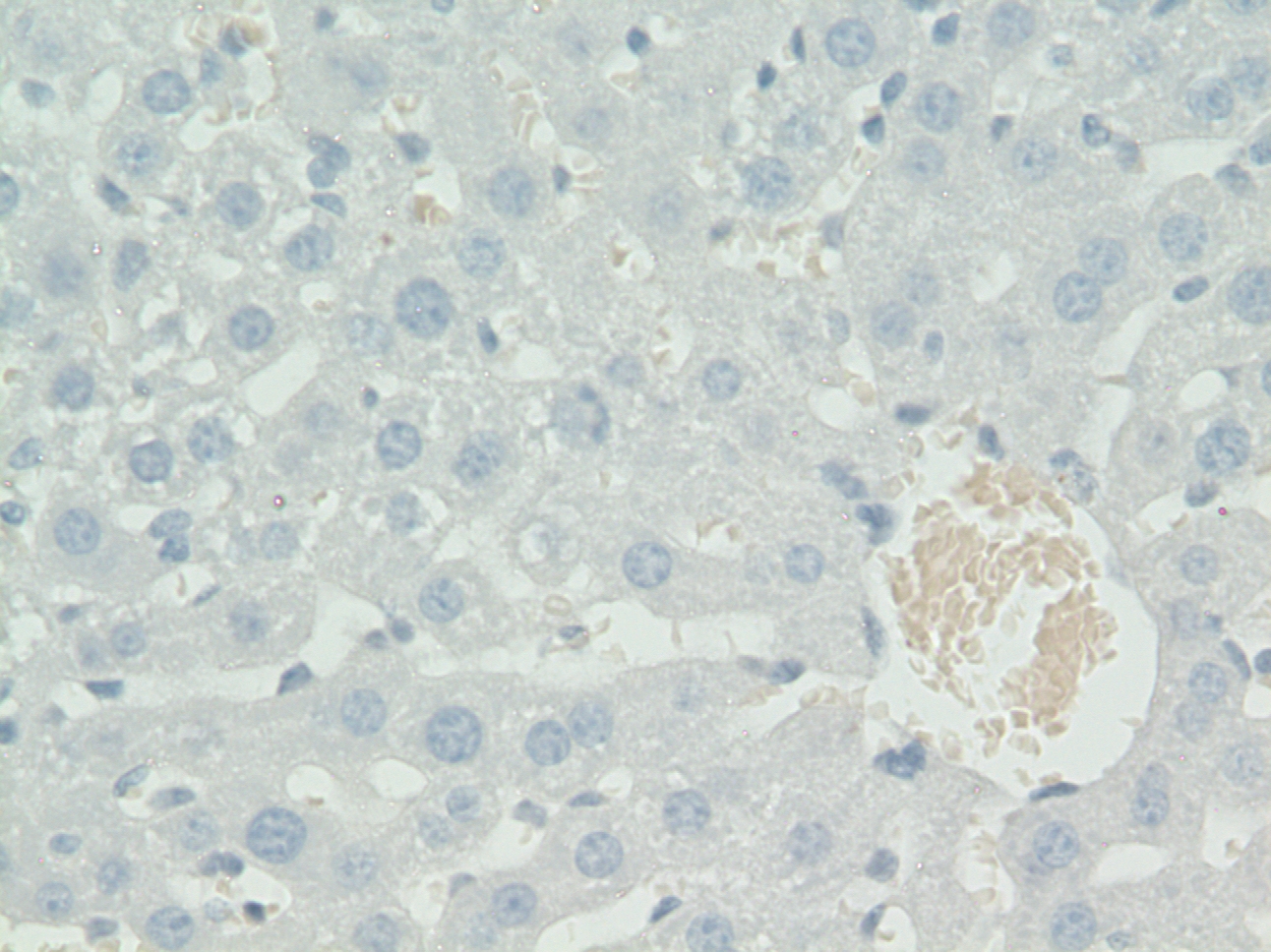

Supplement: Supplementary file 1 [file DataSheet_1.zip › The original images---manuscript 581517/Immunohistochemistry original images/Liver-40x in PBS groups.jpg]

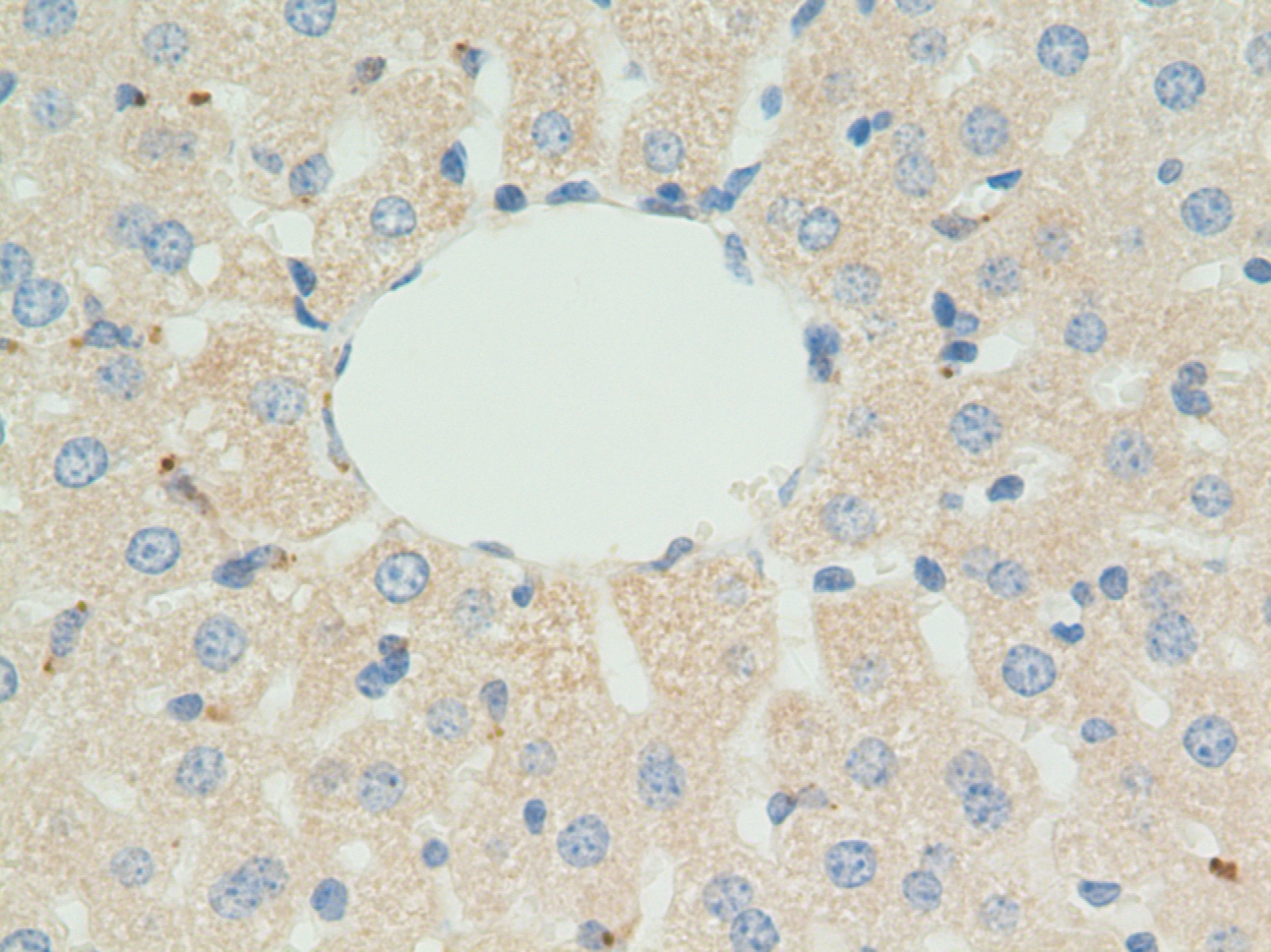

Supplement: Supplementary file 1 [file DataSheet_1.zip › The original images---manuscript 581517/Immunohistochemistry original images/Liver-40x--NLRP3 in mouse infected by S2308.jpg]

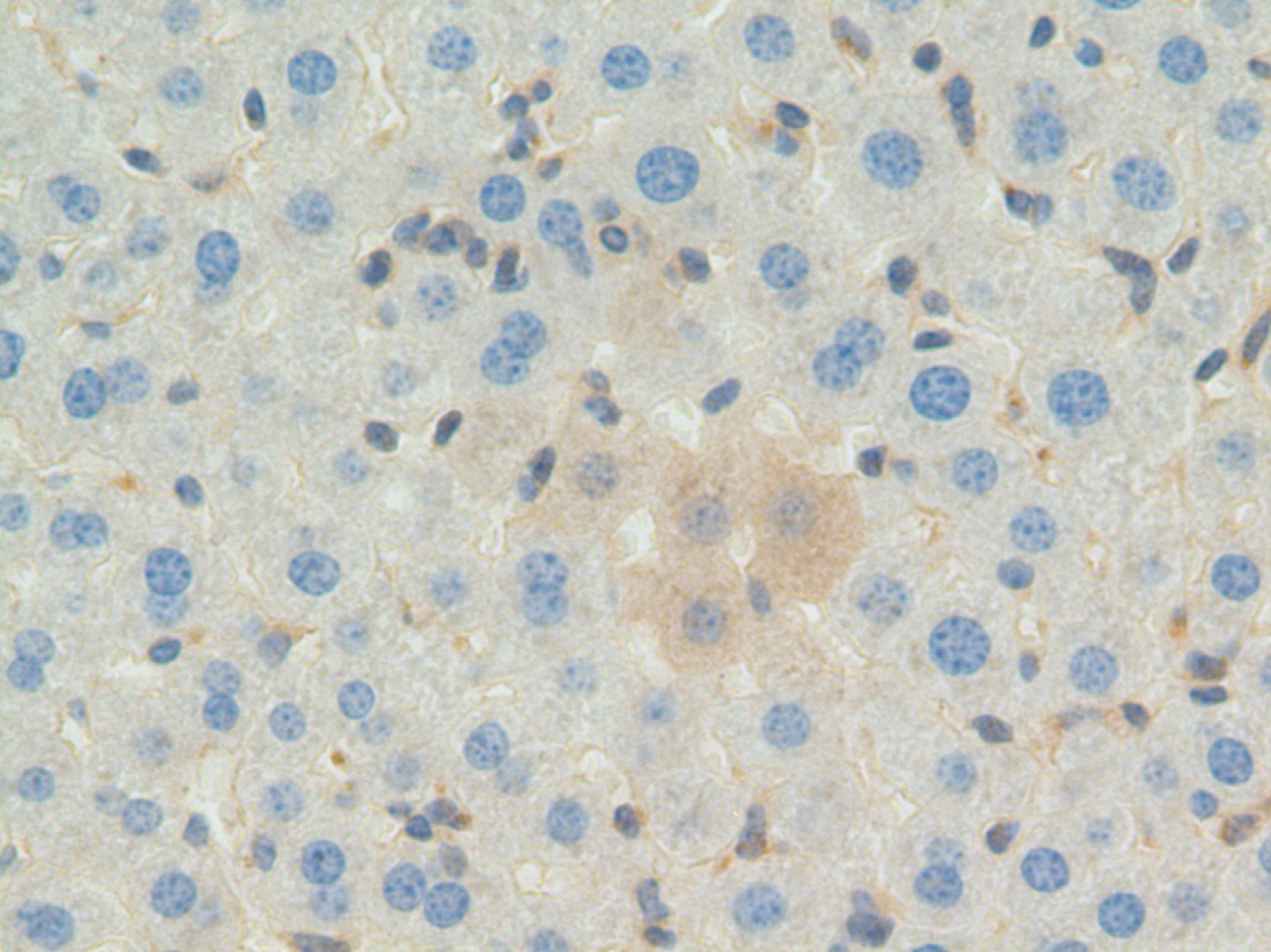

Supplement: Supplementary file 1 [file DataSheet_1.zip › The original images---manuscript 581517/Immunohistochemistry original images/Liverú¡40x--TGF-a┬ in infected by S2308.jpg]

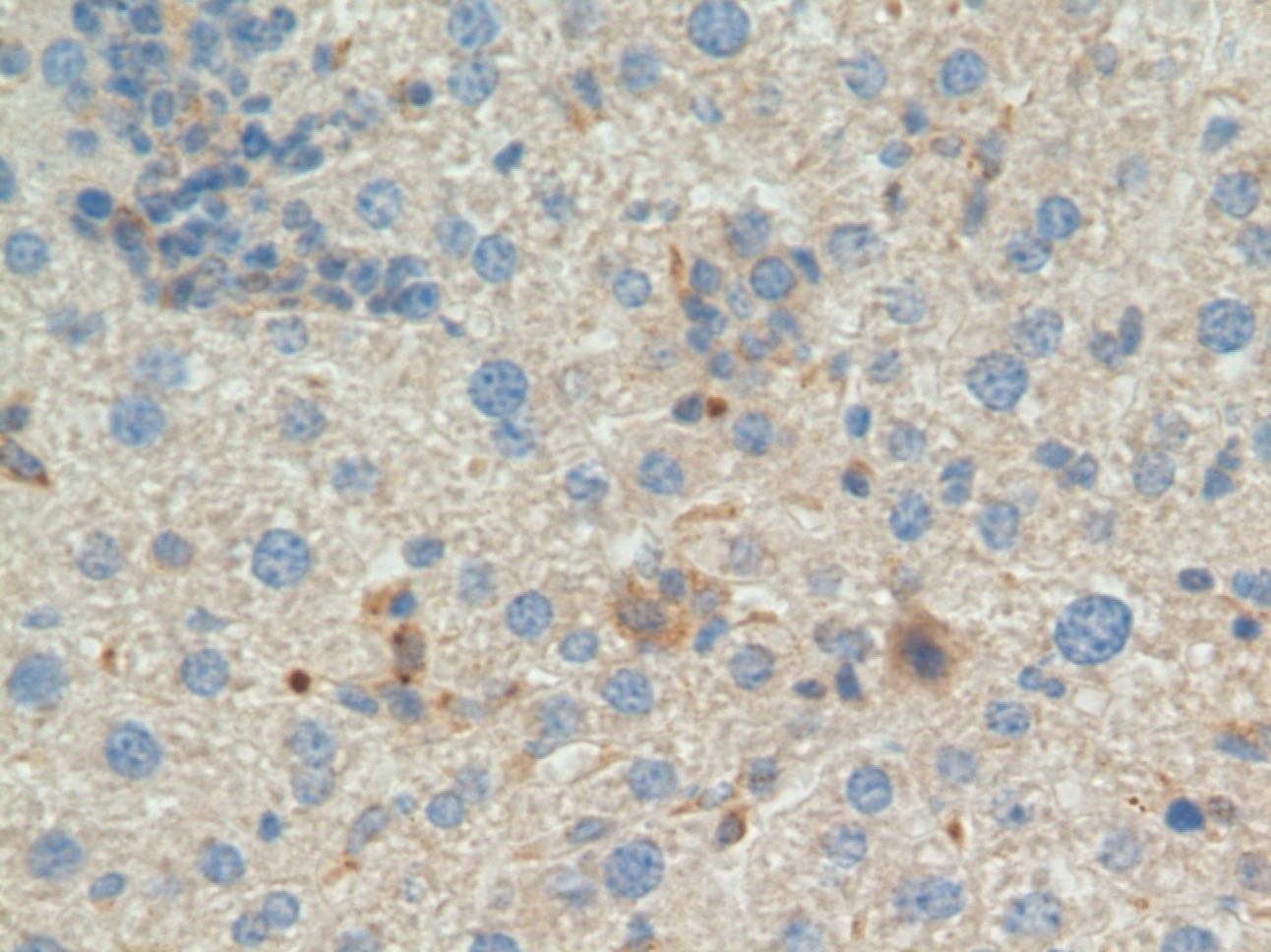

Supplement: Supplementary file 1 [file DataSheet_1.zip › The original images---manuscript 581517/Immunohistochemistry original images/Liverú¡40x--caspase-1 in mouse infected by S2308 (2).jpg]

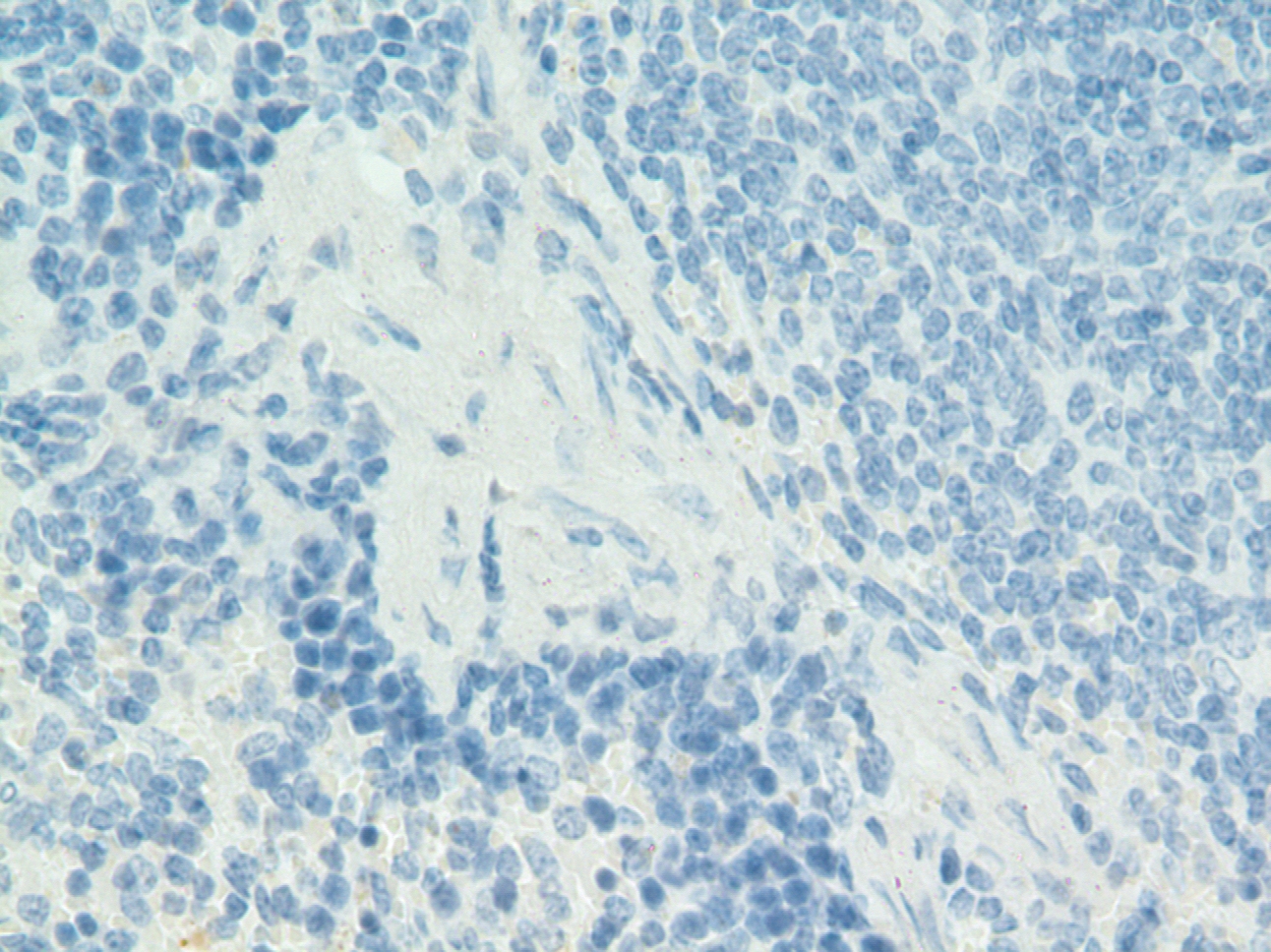

Supplement: Supplementary file 1 [file DataSheet_1.zip › The original images---manuscript 581517/Immunohistochemistry original images/Spleen-40x in PBS groups.jpg]

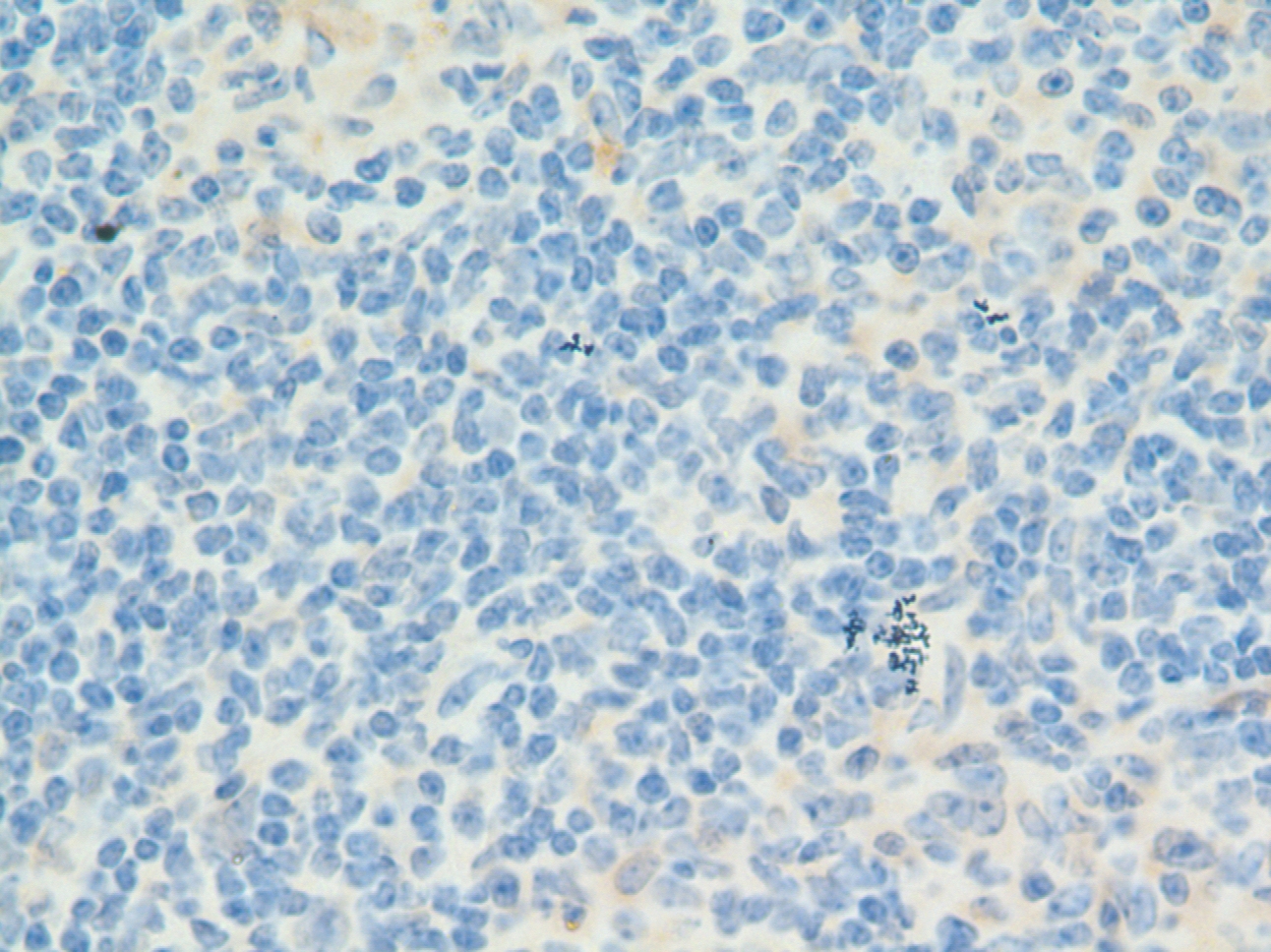

Supplement: Supplementary file 1 [file DataSheet_1.zip › The original images---manuscript 581517/Immunohistochemistry original images/Spleen-40x--NLRP3 in mouse infected by S2308.jpg]

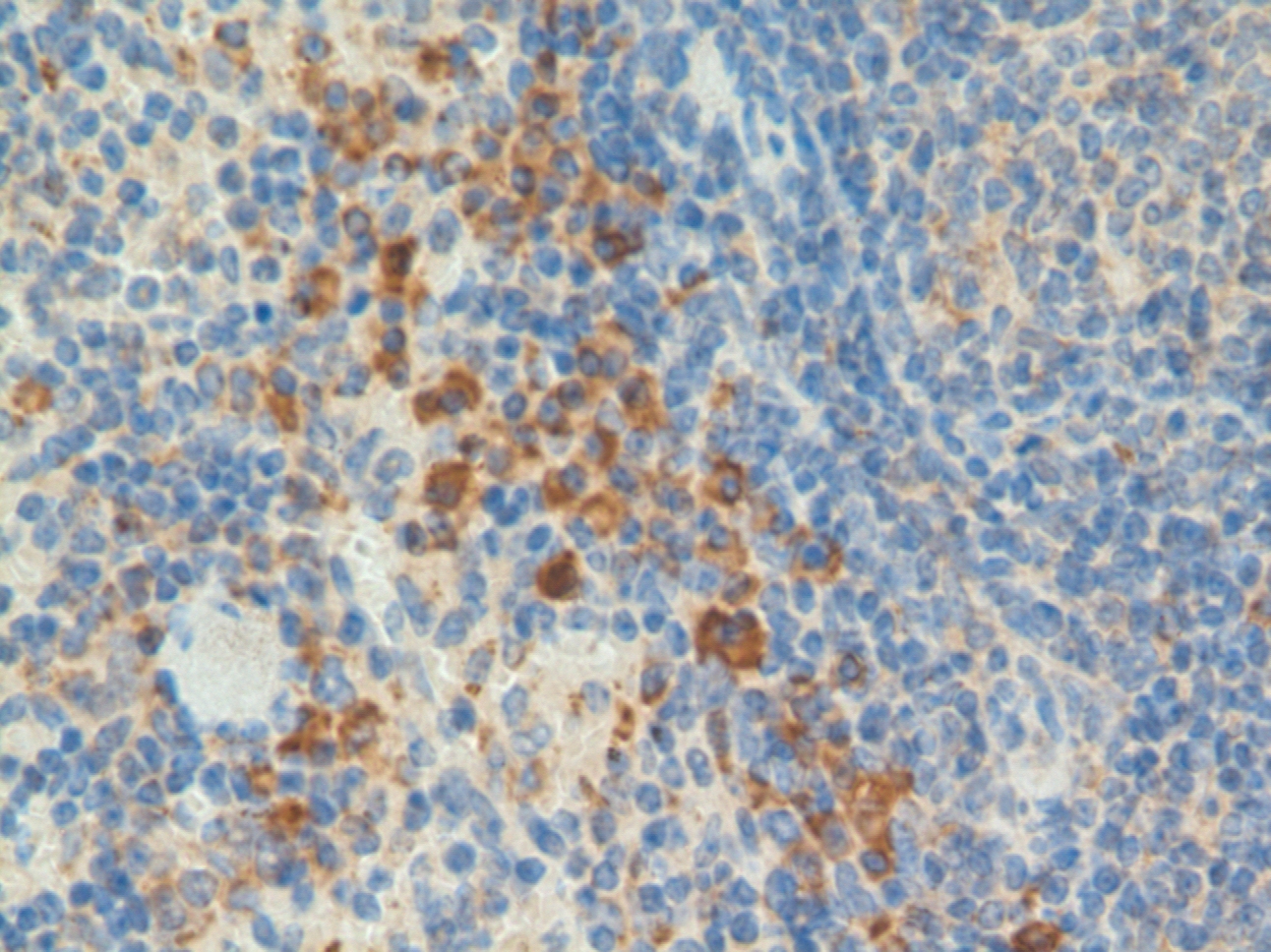

Supplement: Supplementary file 1 [file DataSheet_1.zip › The original images---manuscript 581517/Immunohistochemistry original images/Spleenú¡40x--TGF-a┬ in infected by S2308.jpg]

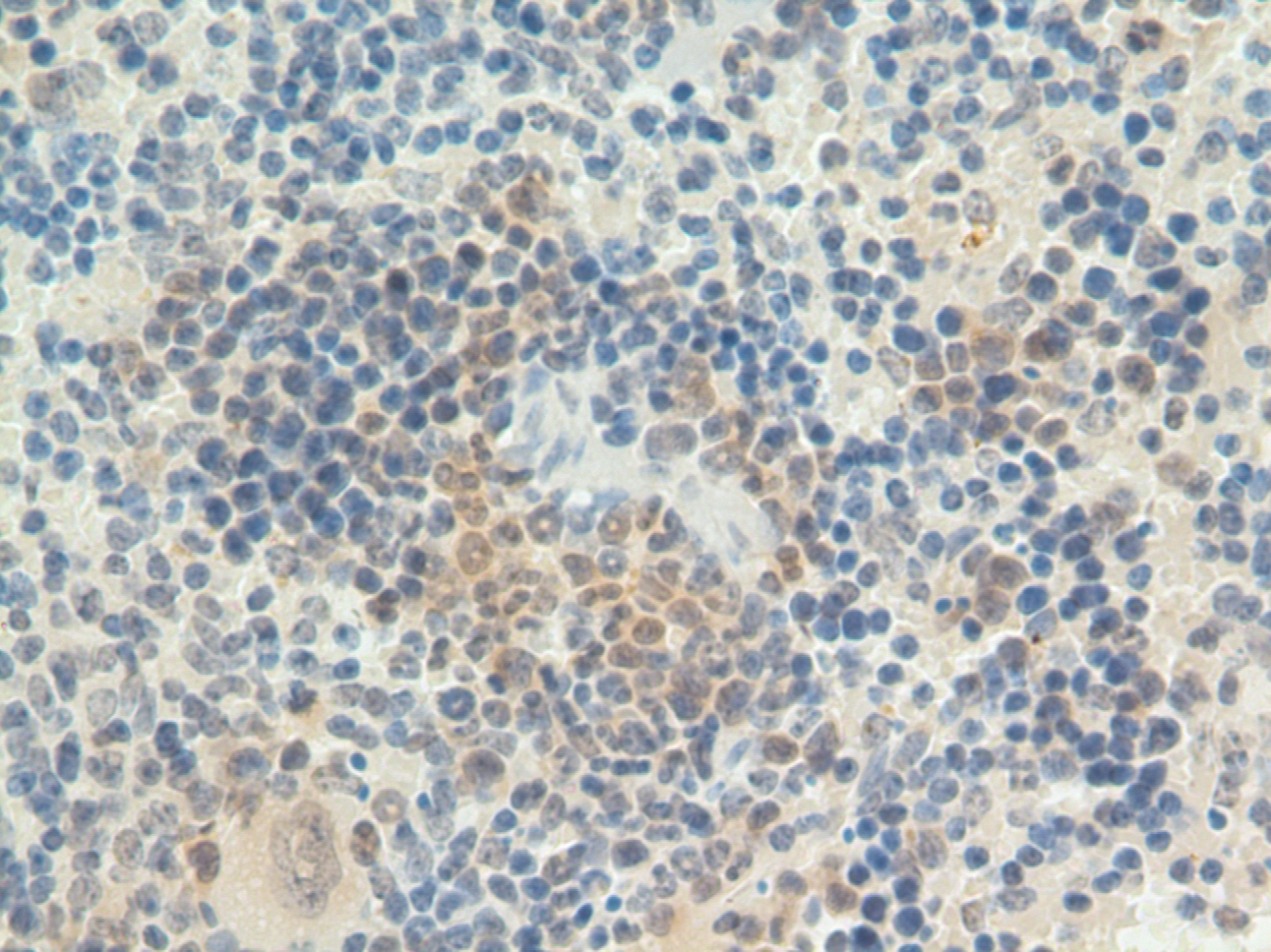

Supplement: Supplementary file 1 [file DataSheet_1.zip › The original images---manuscript 581517/Immunohistochemistry original images/Spleenú¡40x--caspase-1 in mouse infected by S2308 (2).jpg]

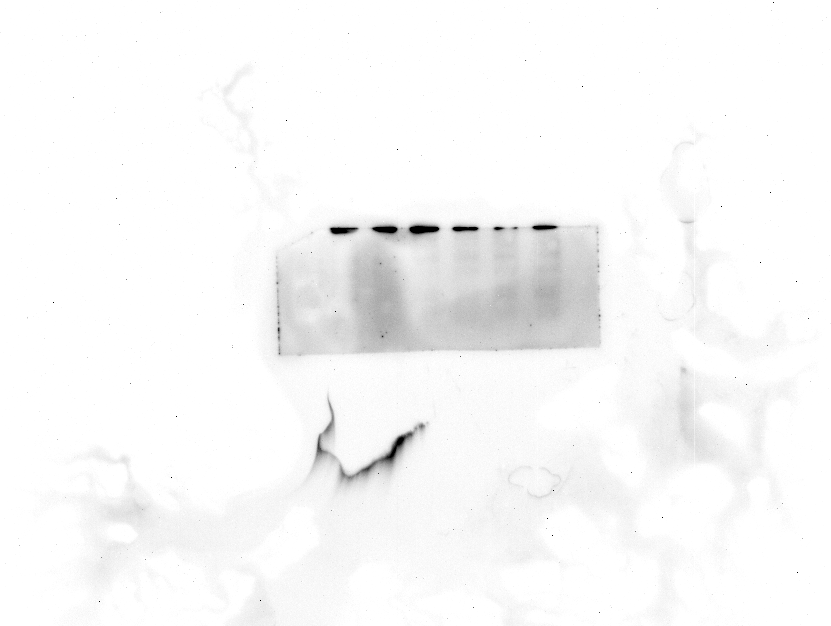

Supplement: Supplementary file 1 [file DataSheet_1.zip › The original images---manuscript 581517/Western blot original images/Figure1 --NLRP3--cropped image for 5 lanes behind(118KD).png]

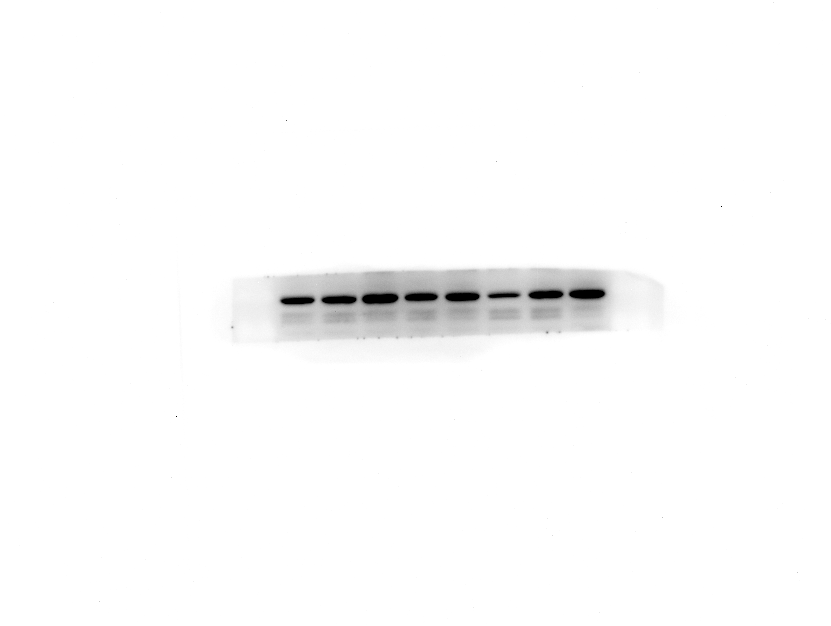

Supplement: Supplementary file 1 [file DataSheet_1.zip › The original images---manuscript 581517/Western blot original images/Figure1 --a┬-actin--cropped image for 5 lanes in front(37KD).png]

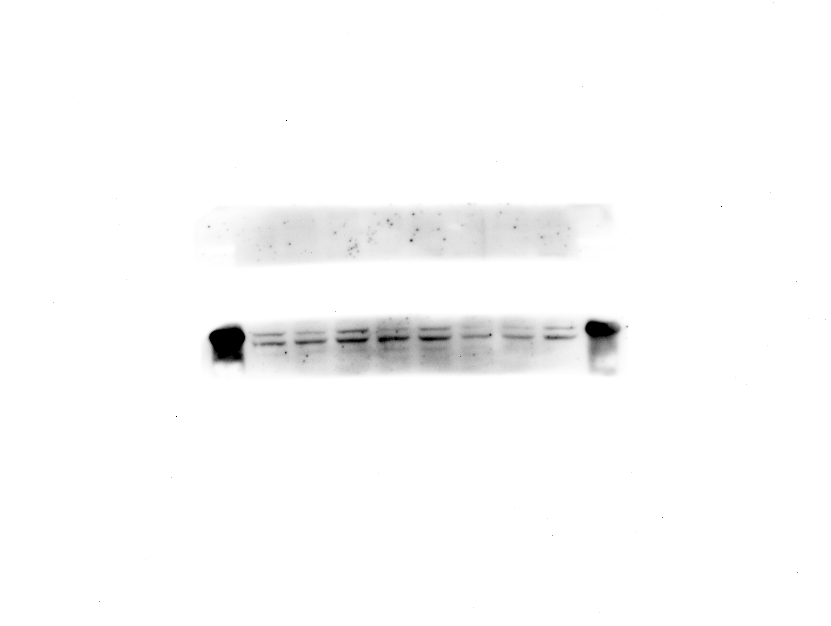

Supplement: Supplementary file 1 [file DataSheet_1.zip › The original images---manuscript 581517/Western blot original images/Figure1--Pro-caspase-1-cropped image for 5 lanes in front (45KD).png]

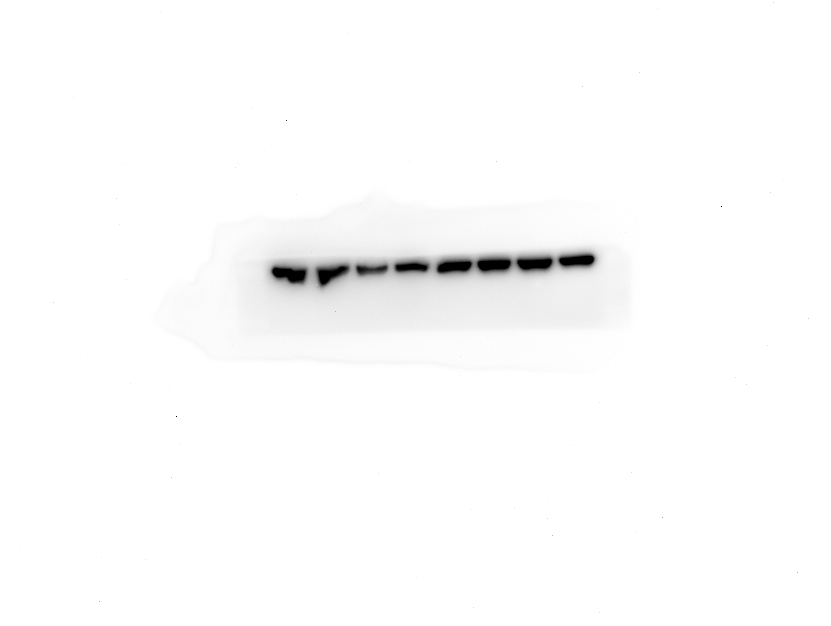

Supplement: Supplementary file 1 [file DataSheet_1.zip › The original images---manuscript 581517/Western blot original images/Figure3 --a┬-actin--cropped image for 6 lanes behind(37KD).jpg]

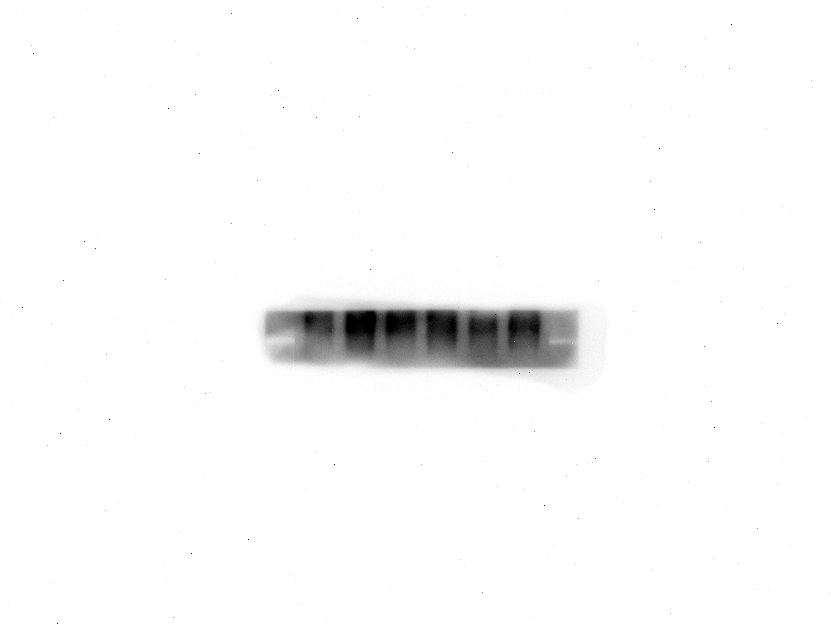

Supplement: Supplementary file 1 [file DataSheet_1.zip › The original images---manuscript 581517/Western blot original images/Figure3--NLRP3 (118KD).jpg]

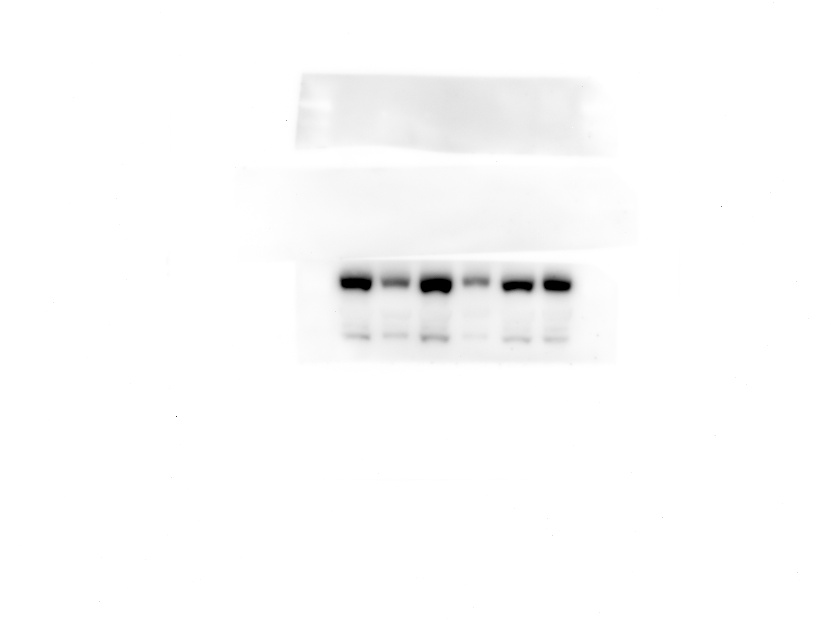

Supplement: Supplementary file 1 [file DataSheet_1.zip › The original images---manuscript 581517/Western blot original images/Figure3--Pro-caspase-1 (45KD).png]

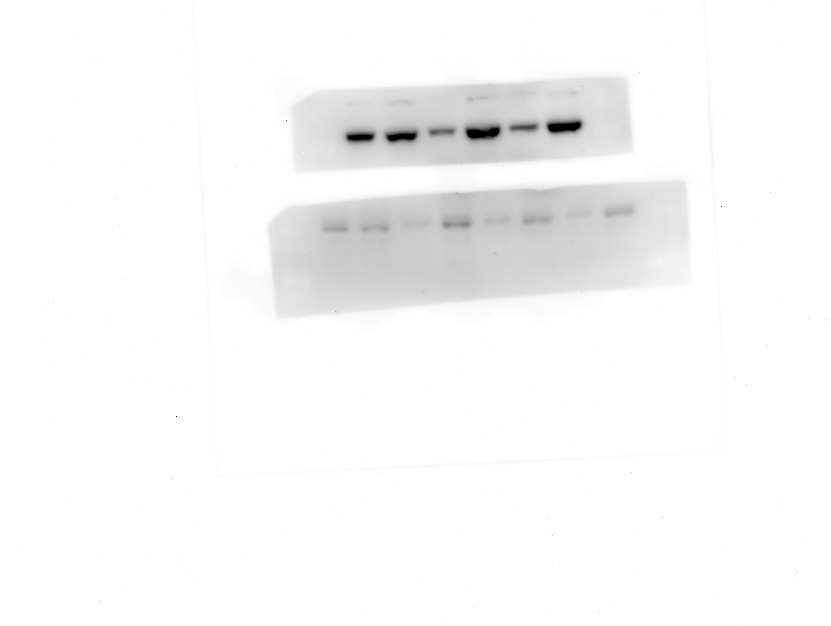

Supplement: Supplementary file 1 [file DataSheet_1.zip › The original images---manuscript 581517/Western blot original images/Figure3--TGF-a┬--cropped the first image including 6 lanes (44KD).jpg]

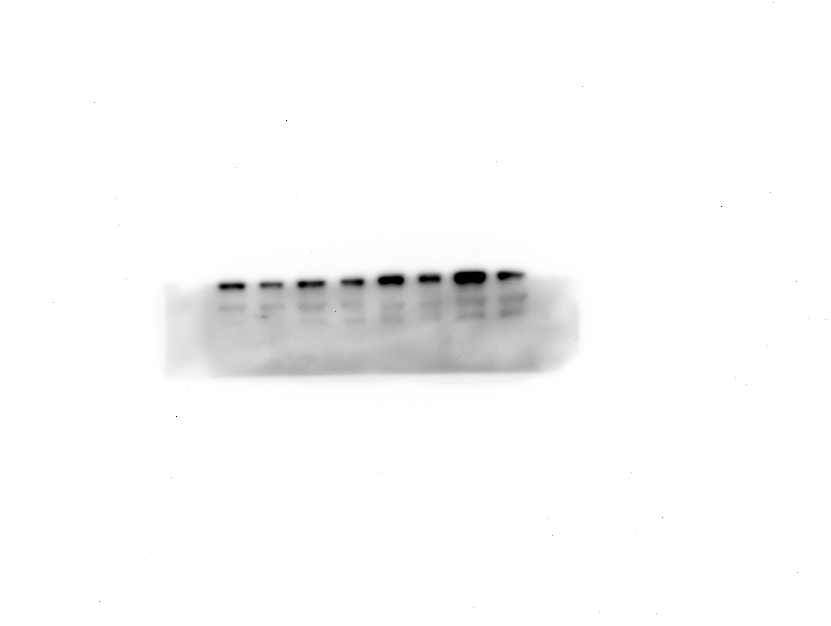

Supplement: Supplementary file 1 [file DataSheet_1.zip › The original images---manuscript 581517/Western blot original images/Figure4 --P65--cropped image for 6 lanes in front (65KD).png]

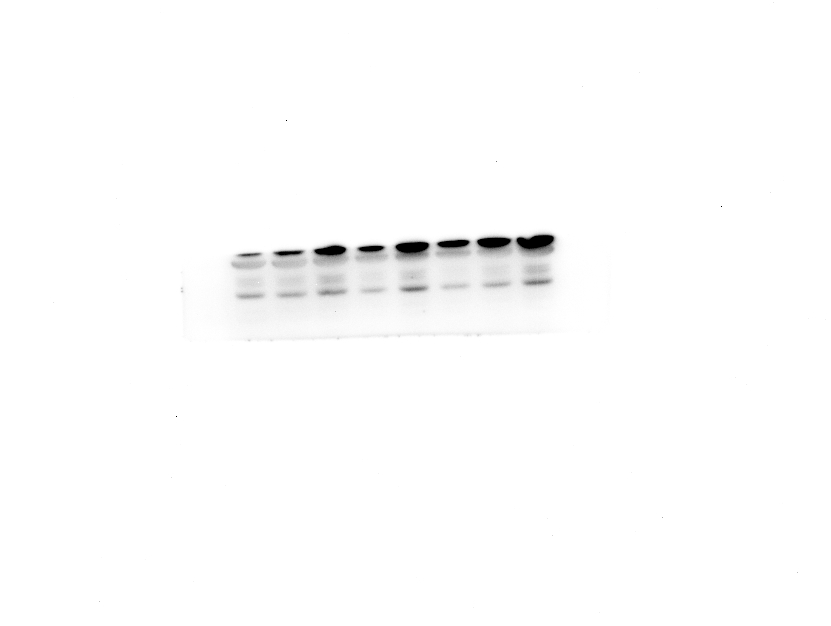

Supplement: Supplementary file 1 [file DataSheet_1.zip › The original images---manuscript 581517/Western blot original images/Figure4 --p-P65--cropped image for 6 lanes in front(65KD).jpg]

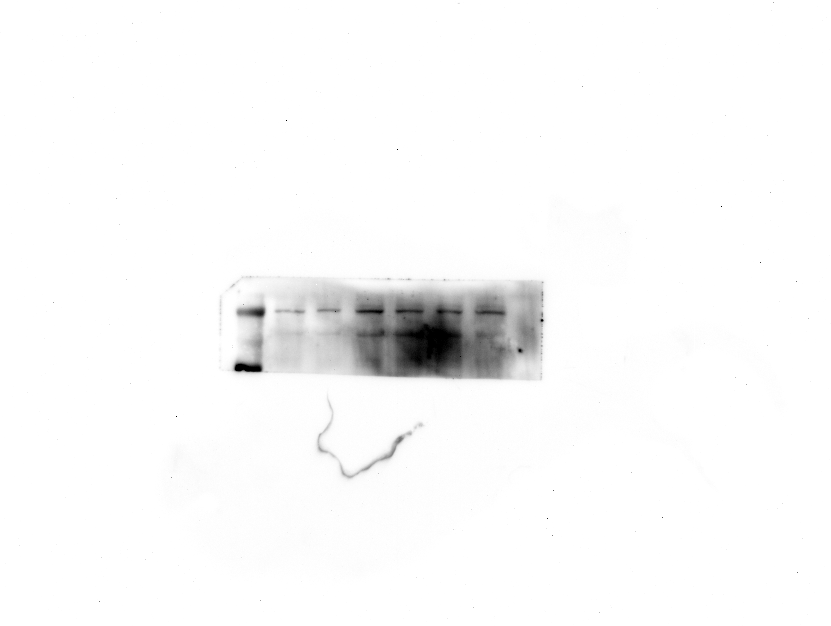

Supplement: Supplementary file 1 [file DataSheet_1.zip › The original images---manuscript 581517/Western blot original images/Figure4 --kB-ras2 (22KD).png]

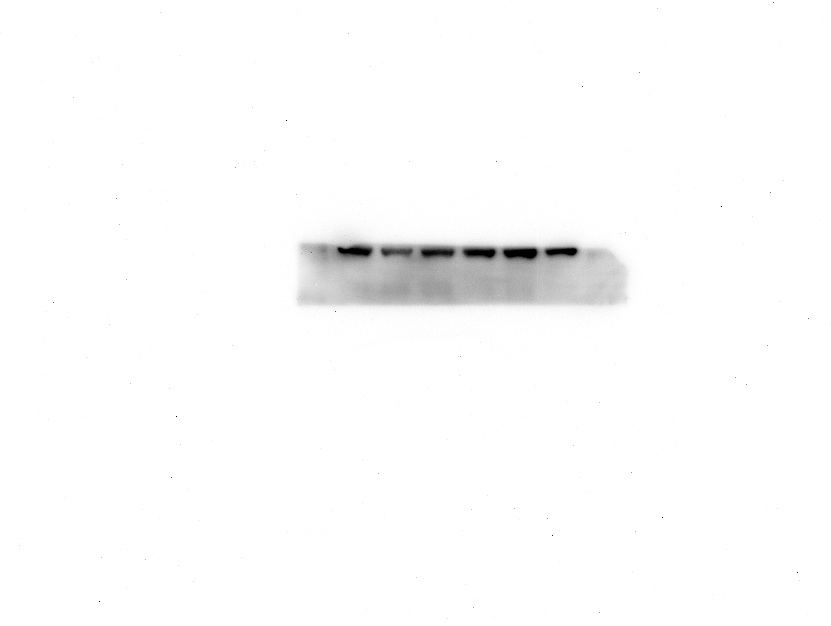

Supplement: Supplementary file 1 [file DataSheet_1.zip › The original images---manuscript 581517/Western blot original images/Figure4 --a┬-actin (37KD).jpg]

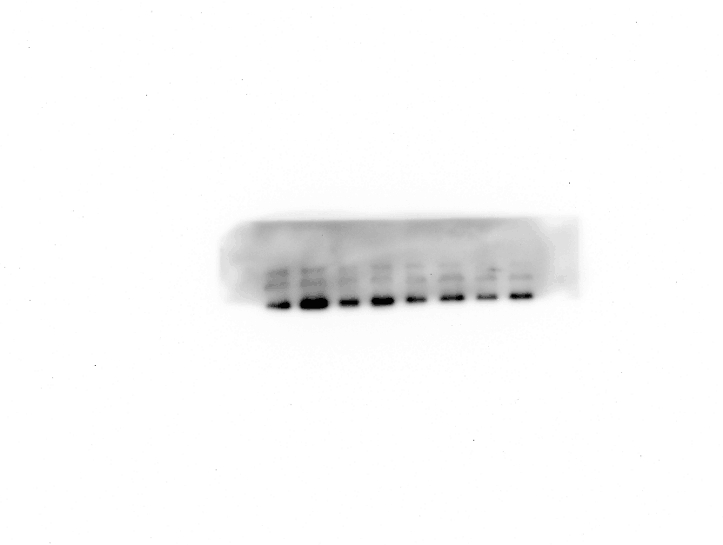

Supplement: Supplementary file 1 [file DataSheet_1.zip › The original images---manuscript 581517/Western blot original images/Figure5 --P65.jpg]

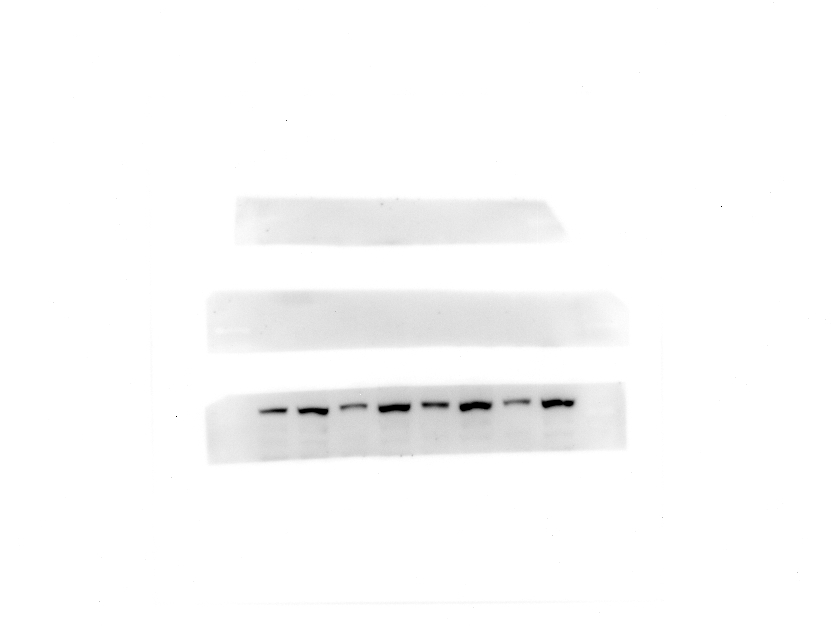

Supplement: Supplementary file 1 [file DataSheet_1.zip › The original images---manuscript 581517/Western blot original images/Figure5 --p-P65.jpg]

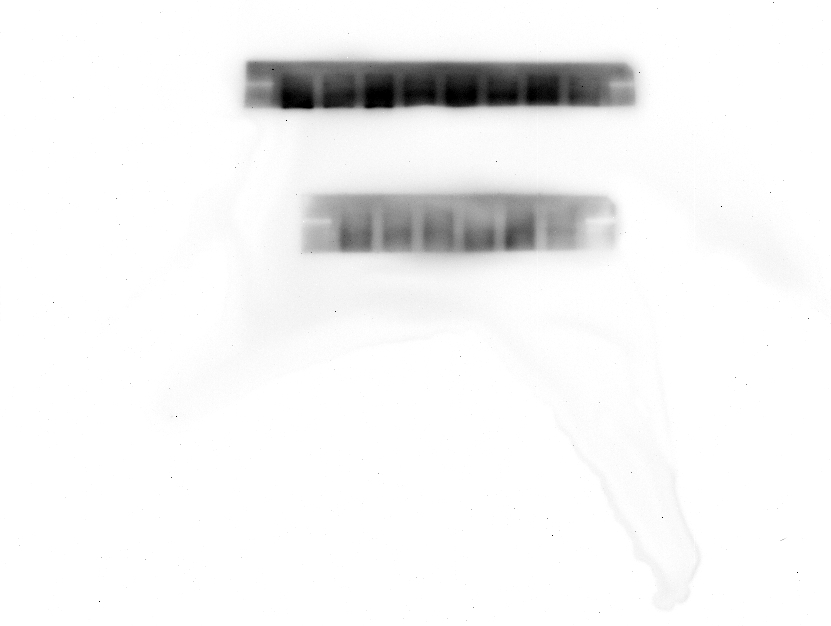

Supplement: Supplementary file 1 [file DataSheet_1.zip › The original images---manuscript 581517/Western blot original images/Figure5 --NLRP3--cropped the first images including 8 lanes.jpg]

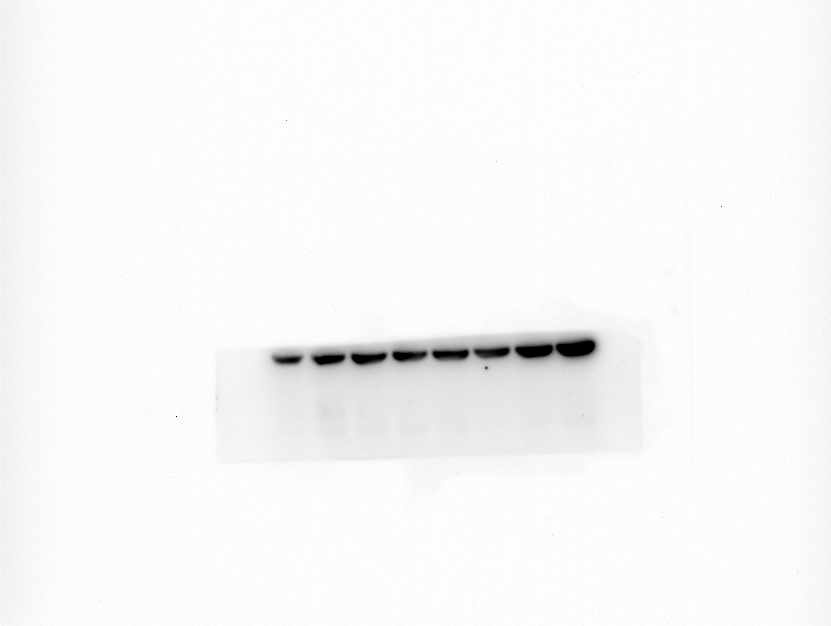

Supplement: Supplementary file 1 [file DataSheet_1.zip › The original images---manuscript 581517/Western blot original images/Figure5 --a┬-actin (37KD).jpg]

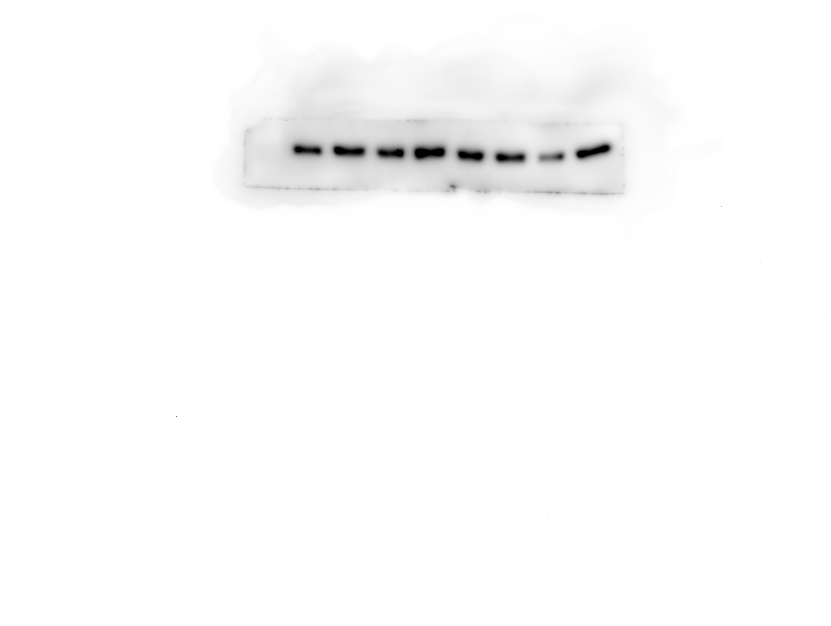

Supplement: Supplementary file 1 [file DataSheet_1.zip › The original images---manuscript 581517/Western blot original images/Figure5--NLRP3(118KD).png]

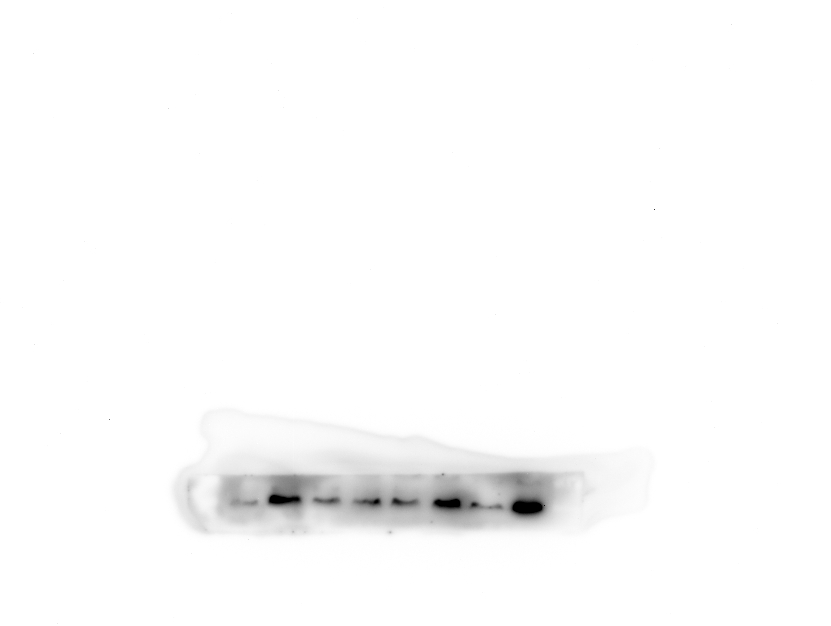

Supplement: Supplementary file 1 [file DataSheet_1.zip › The original images---manuscript 581517/Western blot original images/Figure5--Pro-caspase-1 (45KD).jpg]
