# Supplementary material for: Brucella-Induced Downregulation of lncRNA Gm28309 Triggers Macrophages Inflammatory Response Through the miR-3068-5p/NF-κB Pathway
Source: Front Immunol. 2020 Dec 22;11:581517. doi: 10.3389/fimmu.2020.581517 (PMC7784117; doi:10.3389/fimmu.2020.581517)
Supplement: Supplementary file 2 [file DataSheet_2.pdf]

Supplementary information

Materials and methods

RNA extraction and Real-time Quantitative PCR

The total RNA from cells was extracted using TriZol solution (Invitrogen) according to the manufacturer's instructions. The assessment of the quantity and quality of the total RNA was conducted by a spectrophotometer (NanoDrop Technologies). 1 μg of total RNA was reverse transcribed to cDNA using a Reverse Transcription Kit (Takara Bio). Real-time PCR analyses were performed using SYBR Green-quantitative real-time PCR Master Mix kit (Toyobo Co., Osaka, Japan). the expression of each gene was normalized to GAPDH. The miRNA from cells was extracted using miRcute miRNA Extraction kit (TIANGEN) and miRNA was detected by microRNA Detection Kit (Gene Pharma), miR-3068-5p was normalized to U6. The 2−△△Ct method was used to calculate the fold changes. Each experiment was repeated three times.The primers used are listed in Supplementary Table 1.

Supplementary Table 1. Oligomers Used in This Study

Name	Application	Sequence (5'→3')
qrt-Actin-F	qRT-PCR	GACCTGACTGACTACCTCATGAAGAT
qrt-Actin-R	qRT-PCR	GTCACACTTCATGATGGAGTTGAAGG
qrt-caspase-1-F	qRT-PCR	TCAGCTCCATCAGCTGAAAC
qrt-caspase-1-R	qRT-PCR	TGGAAATGTGCCATCTTCTTT
qrt-GAPDH-F	qRT-PCR	CAGCCTCAAGATCATCAGCA
qrt-GAPDH-R	qRT-PCR	TGTGGTCATGAGTCCTTCCA
qrt-Gm23809-F	qRT-PCR	ATGCTCAACCTCACTGACCG
qrt-Gm23809-R	qRT-PCR	GAGGGGGTACATCCTGGAGT
qrt-IL-18-F	qRT-PCR	GCCATGTCAGAAGACTCTTGCGT
qrt-IL-18-R	qRT-PCR	GTACAGTGAAGTCGGCCAAAGTTGTC
qrt-IL-1β-F	qRT-PCR	TTCCTTGTGCAAGTGTCTGAAG
qrt-IL-1β-R	qRT-PCR	CACTGTCAAAAGGTGGCATTT
qrt-miR-3068-5p -F	qRT-PCR	TGAGGTTGGAGTTCATGCAAGTT
qrt-miR-3068-5p -R	qRT-PCR	CAGTGCGTGTCGTGGAGT
qrt-NLRP3-F	qRT-PCR	AGAAGAGACCACGGCAGAAG
qrt-NLRP3-R	qRT-PCR	CCTTGGACCAGGTTCAGTGT

qrt-p12873-F	qRT-PCR	ACCAGACAGATTTGGGATTTCA
qrt-p12873-R	qRT-PCR	GAATGCCTACTTTCCCGACTAA
qrt-p16218-F	qRT-PCR	CCCAGTCATGCGACTCTCTC
qrt-p16218-R	qRT-PCR	ATGGGGATTTGACCCCAACC
qrt-p30159-F	qRT-PCR	GATAGGAGGCGAGTGAGGAAC
qrt-p30159-R	qRT-PCR	CTGTAAAGGTGACTCGGATGC
qrt-p33714-F	qRT-PCR	CGGGTTTGATTTCTGAGCC
qrt-p33714-R	qRT-PCR	GAGAGAGGAGAGGATGTGCC
qrt-p3852-F	qRT-PCR	TTCTGGGTAGAGCCTCCACTTG
qrt-p3852-R	qRT-PCR	GGAGAAATAGCGTCGTAGTTCAATG
qrt-p65-F	qRT-PCR	TGTGGAGATCATCGAACAGCCG
qrt-p65-R	qRT-PCR	TTCCTGGTCCTGTGTAGCCATTGAT
qrt-p662-F	qRT-PCR	GGGCTCGGTGGTATTGGTGTTGG
qrt-p662-R	qRT-PCR	TTTCCTTGCTTTCTGGATTTGATGACTTG
qrt-TGF-β1-F	qRT-PCR	ATTCCTGGCGTTACCTTGG
qrt-TGF-β1-R	qRT-PCR	AGCCCTGTATTCCGTCTCCT
qrt-U1-F	qRT-PCR	GGCGAGGCTTATCCATTG
qrt-U1-R	qRT-PCR	CCCACTACCACAAATTATGC
qrt-U6-F	qRT-PCR	CAGCACATATACTAAAATTGGAACG
qrt-U6-R	qRT-PCR	ACGAATTTGCGTGTC
miR-3068-5p mimic	mimic	UUGGAGUUCAUGCAAGUUCUAACCUU<br>AGAACUUGCAUGAACUCCAAUU
miR-3068-5p inhibitor	inhibitor	GGUUAGAACUUGCAUGAACUCCAA
p12873-1-antisense	siRNA	UGAUUCUUAAGGCUCAGCCTT
p12873-1-sense	siRNA	GGCUGAGCCUUAAGAAUCATT
p12873-2-antisense	siRNA	AAAGUAUAGAUACGGACUCTT
p12873-2-sense	siRNA	GAGUCCGUAUCUAUACUUUTT
p12873-3-antisense	siRNA	AAACUAGGGCCUUCUAUUCTT
p12873-3-sense	siRNA	GAAUAGAAGGCCCUAGUUUTT
p16218-1-antisense	siRNA	UAUGUGGGAGAAUGAGUGCTT
p16218-1-sense	siRNA	GCACUCAUUCUCCCACAUATT
p16218-2-antisense	siRNA	AAAGGCACUGGAUCCUAGGTT
p16218-2-sense	siRNA	CCUAGGAUCCAGUGCCUUUTT
p16218-3-antisense	siRNA	AAAGACAGCUCUGAGGAGGTT
p16218-3-sense	siRNA	CCUCCUCAGAGCUGUCUUUTT
p30159-1-antisense	siRNA	CACUGGUGUUGUGAGAUGCTT
p30159-1-sense	siRNA	GCAUCUCACAACACCAGUGTT
p30159-2-antisense	siRNA	GUAAAGGUGACUCGGAUGCTT
p30159-2-sense	siRNA	GCAUCCGAGUCACCUUUACTT
p30159-3-antisense	siRNA	AGACGAGGAAAGAACUUCCTT
p30159-3-sense	siRNA	GGAAGUUCUUUCCUCGUCUTT
p33714-1-antisense	siRNA	AUCAGAAGCGAACAAAGGCTT
p33714-1-sense	siRNA	GCCUUUGUUCGCUUCUGAUTT

p33714-2-antisense	siRNA	UUGGCUGCUGGGUUAUUGGTT
p33714-2-sense	siRNA	CCAAUAACCCAGCAGCCAATT
p33714-3-antisense	siRNA	AUACCUCCUCCAUCAAAGCTT
p33714-3-sense	siRNA	GCUUUGAUGGAGGAGGUAUTT
p3852-1-antisense	siRNA	UUAAUGUGUCUAGGUUGUCTT
p3852-1-sense	siRNA	GACAACCUAGACACAUUAATT
p3852-2-antisense	siRNA	UUCUUGGAGAAAUAGCGUCTT
p3852-2-sense	siRNA	GACGCUAUUUCUCCAAGAATT
p3852-3-antisense	siRNA	UUGUAUGCAACAUCGUUGGTT
p3852-3-sense	siRNA	CCAACGAUGUUGCAUACAATT
p662-1-antisense	siRNA	UUUCCCUAGUCCCAAGGUCTT
p662-1-sense	siRNA	GACCUUGGGACUAGGGAAATT
p662-2-antisense	siRNA	AAUCUCUUGCAAAGCAAGCTT
p662-2-sense	siRNA	GCUUGCUUUGCAAGAGAUUTT
p662-3-antisense	siRNA	AAGUUCUCUCUGGAAUGACTT
p662-3-sense	siRNA	GUCAUUCCAGAGAGAACUUTT

Supplementary Table 2 Interfering Efficiency of siRNAs

Name of siRNAs	The relative expression of target LncRNAS（$2^{-\triangle\triangle Ct}$）	Interfering efficiency of siRNAs（$1-2^{-\triangle\triangle Ct}$）
p12873-1	0.14	0.86
p12873-2	0.04	0.96
p16218-1	0.21	0.79
p16218-2	0.23	0.77
p16218-3	0.33	0.67
p17484-3	0.44	0.56
p30159-1	0.2	0.8
p30159-2	0.85	0.15
p30159-3	0.35	0.65
p33714-1	0.02	0.98
p33714-2	0.09	0.91
p33714-3	0.01	0.99
p3852-1	0.33	0.67
p3852-2	0.53	0.47
p3852-3	1	0
p662-1	0.2	0.8
p662-2	0.19	0.81
p662-3	0.12	0.88

Supplementary figures

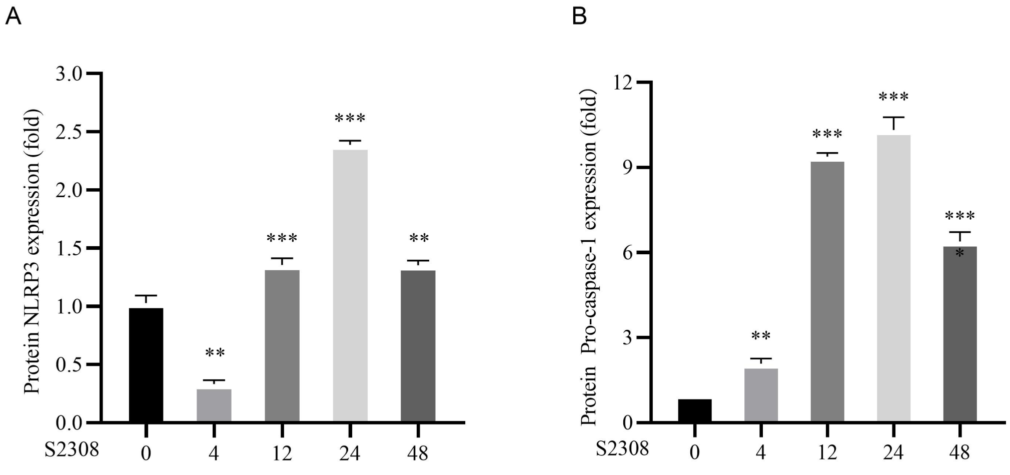

Supplementary Figure 1. The quantitive graph of relative protein expression levels of inflammatory proteins at different time of S2308 . (**A**) The quantitive graph of relative protein expression levels of NLRP3 at 4h, 12h, 24h and 48h of S2308 infection. (**B**) The quantitive graph of relative protein expression levels of NLRP3 at 4h, 12h, 24h and 48h of S2308 infection

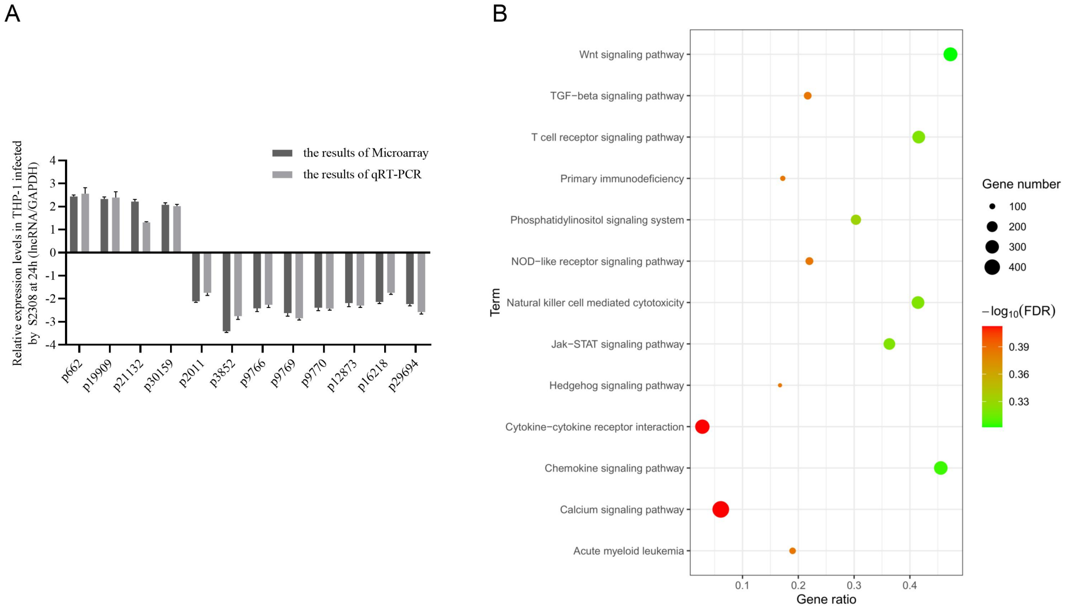

Supplementary Figure 2. Analysis of RNA Sequencing for THP-1 cells infected by S2308. (**A**) The expressions of partial lncRNAs in THP-1 cells infected by S2308 for 24h were verified using qRT-PCR. (**B**) KEGG enrichment analysis of differentially expressed lncRNAs in THP-1 cells infected by S2308 for 24h. .

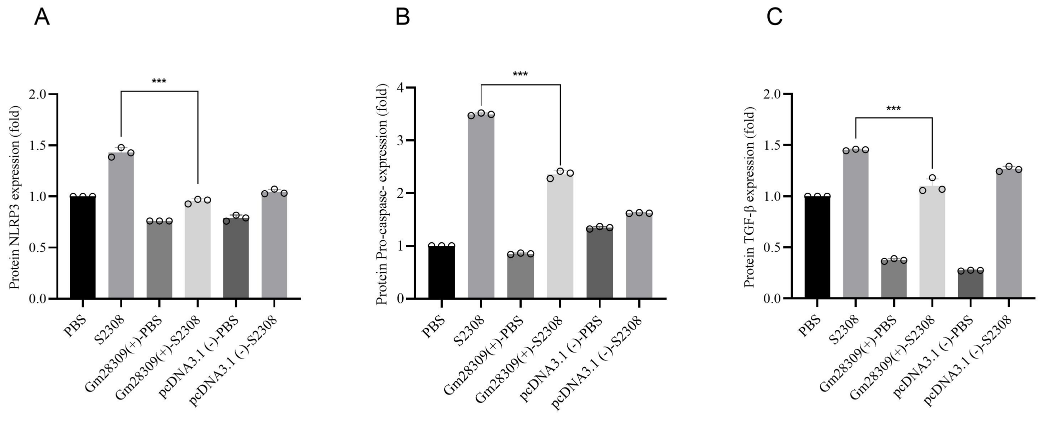

Supplementary Figure 3. The quantitive graph of relative protein expression levels of inflammatory proteins while overexpressed Gm28309 in RAW264.7 cells. (**A-C**) The quantitive graph of relative protein expression levels of NLRP3, Pro-caspase-1 and TGF-β respectively while overexpressed Gm28309 in RAW264.7 cells.following 24 S2308 infection.

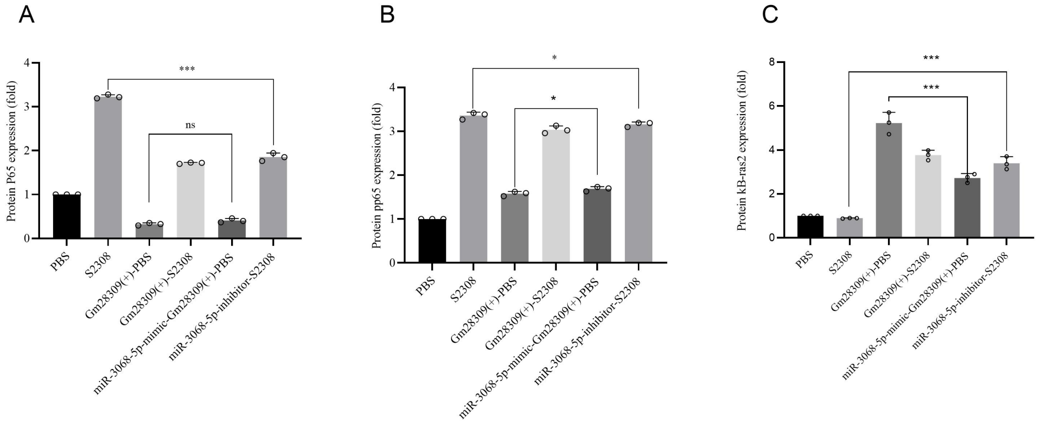

Supplementary Figure 4. The quantitive graph of relative protein expression levels of NF-κB signaling pathway while up or down-expressed miR-3068-5p in RAW264.7 cells. (**A-C**) The quantitive graph of relative protein expression levels of P65, pp65 and κB-Ras2 respectively while overexpressed Gm28309 in RAW264.7 cells.following 24 S2308 infection.

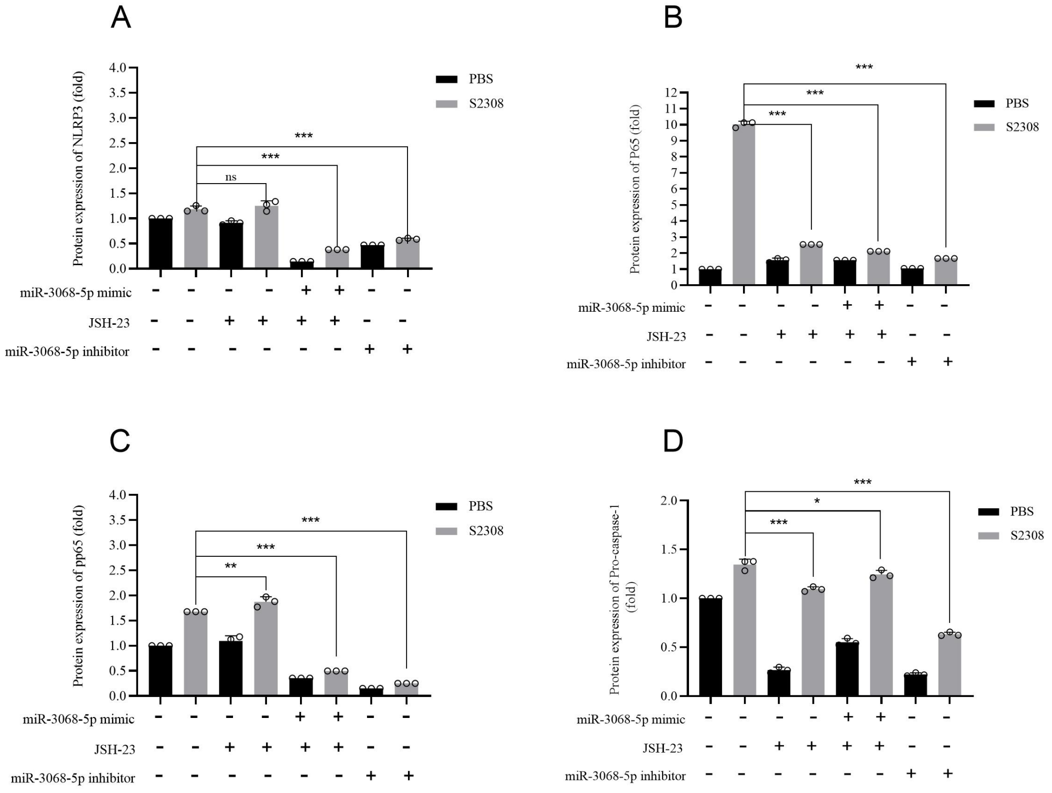

Supplementary Figure 5. The quantitive graph of relative protein expression levels of inflammatory and NF-κB signaling pathway while up or down-expressed miR-3068-5p or inhibited p65 in RAW264.7 cells. (A-D) The quantitive graph of relative protein expression levels of NLRP3, Pro-caspase-1, P65 and pp65 respectively while up or down-expressed miR-3068-5p or inhibited p65 in RAW264.7 cells.
