# Supplementary material for: Brucella-Induced Downregulation of lncRNA Gm28309 Triggers Macrophages Inflammatory Response Through the miR-3068-5p/NF-κB Pathway
Source: Front Immunol. 2020 Dec 22;11:581517. doi: 10.3389/fimmu.2020.581517 (PMC7784117; doi:10.3389/fimmu.2020.581517)
Supplement: Supplementary file 1 [file DataSheet_1.zip › The original images---manuscript 581517/Western blot original images/Annotation for original images.doc]

**Annotation for original images for western blot**

Figure1, NLRP3(118KD), we cropped image for 5 lanes behind from the original image

Figure1,Pro-caspase-1(45KD), we cropped image for 5 lanes in front from the original image;

Figure1, β-actin(37KD), we cropped image for 5 lanes in front from the original image;

Figure3, β-actin(37KD), we cropped image for 6 lanes behind from the original image;

Figure3,TGF-β(44KD), we cropped the first images including 6 lanes for the ideal image;

Figure4,P65(65KD), we cropped image for 6 lanes in front from the original image;

Figure4,p-P65(65KD),we cropped image for 6 lanes in front the original image;

Figure4,kB-ras2(22KD),we cropped image for 6 lanes in upper line from the original image;

Figure5,NLRP3(118KD),we cropped the first images including 8 lanes;
